# Modelling chemotherapy effects on granulopoiesis

**DOI:** 10.1186/s12918-014-0138-7

**Published:** 2014-12-24

**Authors:** Sibylle Schirm, Christoph Engel, Markus Loeffler, Markus Scholz

**Affiliations:** Institute for Medical Informatics, Statistics and Epidemiology (IMISE) Medical Faculty, University of Leipzig, Haertelstraße 16-18, 04107 Leipzig, Germany; LIFE Research Center, Leipzig, Germany

**Keywords:** Leukopenia, G-CSF, Filgrastim, Pegfilgrastim, Chemotherapy

## Abstract

**Background:**

Although the growth-factor G-CSF is widely used to prevent granulotoxic side effects of cytotoxic chemotherapies, its optimal use is still unknown since treatment outcome depends on many parameters such as dosing and timing of chemotherapies, pharmaceutical derivative of G-CSF used and individual risk factors. We showed in the past that a pharmacokinetic and –dynamic model of G-CSF and human granulopoiesis can be used to predict the performance of yet untested G-CSF schedules. However, only a single chemotherapy was considered so far.

In the present paper, we propose a comprehensive model of chemotherapy toxicity and combine it with our cell kinetic model of granulopoiesis. Major assumptions are: proportionality of cell numbers and cell loss, delayed action of chemotherapy, drug, drug-dose and cell stage specific toxicities, no interaction of drugs and higher toxicity of drugs at the first time of application. Correspondingly, chemotherapies can be characterized by a set of toxicity parameters which can be estimated by fitting the predictions of our model to clinical time series data of patients under therapy. Data were either extracted from the literature or were received from cooperating clinical study groups.

**Results:**

Model assumptions proved to be feasible in explaining granulotoxicity of 10 different chemotherapeutic drugs or drug-combinations applied in 33 different schedules with and without G-CSF. Risk groups of granulotoxicity were traced back to differences in toxicity parameters.

**Conclusion:**

We established a comprehensive model of combined G-CSF and chemotherapy action in humans which allows us to predict and compare the outcome of alternative G-CSF schedules. We aim to apply the model in different clinical contexts to optimize and individualize G-CSF treatment.

**Electronic supplementary material:**

The online version of this article (doi:10.1186/s12918-014-0138-7) contains supplementary material, which is available to authorized users.

## Background

The effectivity of antineoplastic chemotherapy of some cancer types, such as lymphomas or breast cancer, depends on dose density of applied cytostatic drugs [1-6]. Dose density is defined as the amount of drug given per body surface per time unit (mg/m^2^/week) [7,8]. It has been shown that a decrease in dose density such as treatment delays or dose reductions, can have negative impact on remission rates, recurrence rates and overall survival rates [9-15].

Physicians are frequently forced to reduce dose density due to serious chemotherapy-associated side effects, of which neutropenia, i.e. a reduction of white blood cells, is the most common one. Because neutrophils are an essential part of the nonspecific immune system, neutropenic patients are prone to bacterial and fungal infections, frequently resulting in an increased need of antibiotics, prolonged hospitalization and a higher risk of therapy discontinuation [16-22].

To ameliorate neutropenia, the recombinant haematopoietic growth factor G-CSF (granulocyte colony stimulating factor) is routinely applied. It is a major requirement to make dose-dense therapies feasible. G-CSF increases the mitotic activity, accelerates the maturation of different immature granuloid precursor cells in the bone marrow and increases the release of mature granulocytes into blood [23,24]. Nowadays, a variety of pharmaceutical derivatives of G-CSF are available differing in pharmacokinetic and –dynamic properties. In consequence, combined chemotherapy and G-CSF treatment result in complex dynamics of granulocytes due to the interaction of G-CSF pharmacokinetics, G-CSF induced granulocytosis via different mechanisms and chemotherapy induced cell destruction.

We recently established a biomathematical model of G-CSF applications in humans explaining numerous scenarios of G-CSF applications of the most frequently used derivatives Filgrastim and Pegfilgrastim into healthy volunteers and first simple chemotherapies [25]. However, the large variety of chemotherapies supported by G-CSF in current clinical practice is not yet covered.

In the present article we introduce a substantially refined model of chemotherapy action applicable for different diseases and risk groups. The major objective of our model is to allow predictions of the dynamics of granulocytes after combined chemotherapy and G-CSF applications especially regarding effects of alternative, yet untested G-CSF treatment schedules on neutropenia.

## Methods

We recently developed a comprehensive model of pharmacokinetics and pharmacodynamics of Filgrastim and Pegfilgrastim [25]. We briefly sketch this model in the following:

### PK/PD model structure and basic properties

**Figure 1:**
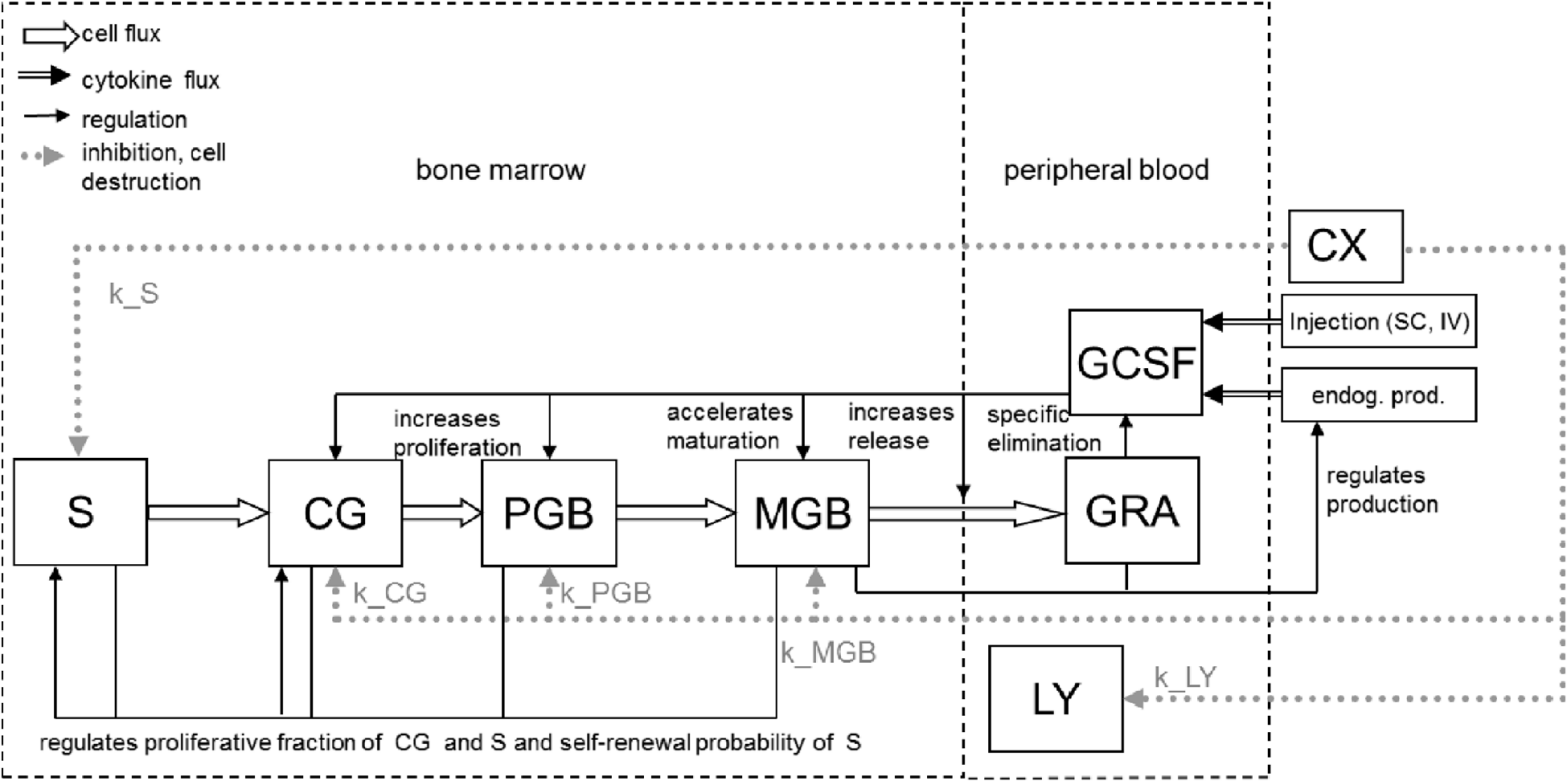
**Structure of the granulopoiesis model under chemotherapy and G-CSF treatment.** Boxes represent major cell- or cytokine compartments of the model: S = haematopoietic stem cells, CG = granulopoietic progenitors, PGB = granulopoietic precursors, MGB = maturing granulopoietic precursors in bone marrow, GRA = mature granulocytes in blood, LY = lymphocytes. We modelled two G-CSF derivatives (fil = Filgrastim, peg = Pegfilgrastim). Arrows represent cell/cytokine fluxes and interactions. CX represents the strength of chemotherapy (see below).

Figure 1 depicts the structure of the PK/PD-model. Granulopoiesis is divided into five distinct cell compartments, representing haematopoietic stem cells (S), early progenitors (CG), proliferating precursors (PGB), maturing precursors (MGB) and mature granulocytes in circulation (GRA). Dynamics of each compartment are described by ordinary differential equations (ODE). The system is regulated by several feed-back loops, mostly mediated by the cytokine G-CSF. Endogenous G-CSF production is regulated by the cell demand of the system.

All granulopoietic cells originate from the stem cell compartment. Cell division and differentiation rates in S are regulated in such a way that self-maintenance gains priority in case of low stem cell numbers. Cells committed to granulopoietic lineages enter the subsequent compartment CG, which represents the most immature cell stage committed to granulopoiesis. The next compartment, PGB, represents the mitotic granuloid precursors (myeloblasts, promyelocytes and myelocytes). Compartment MGB represents all non-mitotic precursors (metamyelocytes, banded cells and mature neutrophils) in the bone marrow. The final compartment, GRA, comprises all mature neutrophils in the peripheral blood. Reductions of lymphocytes LY due to chemotherapy are also modelled.

Without chemotherapy, changes of compartment sizes are determined by balance equations of cell influx, cell production and cell efflux or degradation:

$$ \frac{dC(t)}{dt}={C}_{in}(t)\ast A(t)-\frac{C(t)}{T(t)} $$

where $\frac{dC(t)}{dt}$ represents the changing rate of compartment size, *C*_*in*_(*t*) represents the cell influx rate from the preceding compartment, *A*(*t*) the amplification of cell numbers and *T*(*t*) is the average time of a cell residing in the compartment (transition time).

Filgrastim and Pegfilgrastim are assumed to have different pharmacodynamic properties in the model, whereas Filgrastim and endogenous G-CSF are assumed to be undistinguishable. Amplification rate and transition time in PGB increase with G-CSF serum concentration. In contrast, the transition time in MGB and the apoptosis rate are reduced with increasing G-CSF concentrations. Details of Filgrastim and Pegfilgrastim pharmacokinetics, pharmacodynamics, and corresponding regulatory processes are described elsewhere [25].

On the basis of this baseline model, we aim at developing a more comprehensive model of cytotoxic chemotherapy action on granulopoiesis. Corresponding assumptions and equations are explained in the following. Some detailed information can be found in [26].

### Modeling chemotherapy

Next we present and discuss our assumptions reagarding chemotherapy modelling in detail.

### Assumption 1 (injection)

Injection of chemotherapy is modeled by pulse functions according to the applied dosing and timing schedules. In general, each drug is modeled by a separate pulse function, where the length of the pulse corresponds to the injection time and the amplitude is normalized in such a way that the area under the curve after a single injection equals one (see equations below).

### Assumption 2 (delayed action)

We assumed a delayed maximum of chemotherapy damage after injection. This is modeled in a phenomenological rather than mechanistic way by a set of concatenated first order transitions resulting in a delayed maximum after injection (see [27]).

$$ \frac{{\mathrm{d}\Psi}_{\mathrm{d}\mathrm{rug}}^{\left(\mathrm{i}\right)}\left(\mathrm{t}\right)}{\mathrm{d}\mathrm{t}}={\Psi}_{drug\_ out}^{\left(i-1\right)}\ \left(\mathrm{t}\right)-{\mathrm{k}}_{\mathrm{Delay}}^{\mathrm{d}\mathrm{rug}}{\Psi}_{\mathrm{tox}}^{\mathrm{i}}\left(\mathrm{t}\right),\kern0.5em i=1,\dots, 4,\mathrm{with} $$

$$ {\Psi}_{drug}^{(0)}\left(\mathrm{t}\right)={\mathrm{CHEMO}}^{\mathrm{drug}}\left(\mathrm{t}\right), $$

$$ {\mathrm{CHEMO}}^{\mathrm{drug}}\left(\mathrm{t}\right)={\displaystyle \sum_{i=1}^{{\displaystyle {N}^{cycle}}}\left(\mathrm{Hv}\right(\mathrm{t}-{\mathrm{t}}_{\mathrm{i}}}\left)-\mathrm{Hv}\left(\mathrm{t}-{\mathrm{t}}_{\mathrm{i}}-{\mathrm{t}}_{\inf}\right)\right)/{\mathrm{t}}_{\inf } $$

$$ {\Psi}_{drug\_ out}^{(i)}\ \left(\mathrm{t}\right)={\mathrm{k}}_{\mathrm{Delay}}^{\mathrm{drug}}{\Psi}_{drug}^{(i)}\ \left(\mathrm{t}\right),\kern0.5em i=1,\dots, 4, $$

where Hv is the Heavyside function: $H\mathrm{v}\ \left(\mathrm{t}\right)=\left\{{}_{1\ :\ \mathrm{t}>0}{}^{0\ :\ \mathrm{t}\le 0}\right.,\kern0.5em {\mathrm{t}}_{\mathrm{i}}$ are the time points of chemotherapy applications and t_inf_ is the infusion time. Thus, function CHEMO represents the chemotherapy schedule. In summary, ${\Psi}_{\mathrm{drug}\_\mathrm{out}}^{(4)}\left(\mathrm{t}\right)$ represents the strength of the (delayed) toxic effect.

### Assumption 3 (toxicity)

Drug, drug-dose and cell-stage specific toxicity functions are derived from ${\Psi}_{\mathrm{drug}\_\mathrm{out}}^{(4)}\left(\mathrm{t}\right)$ by multiplications with specific toxicity values: ${\mathrm{K}}_{\mathrm{X}}^{\mathrm{drug}}(t)$

$$ {\Psi}_{\mathrm{drug}}^{\mathrm{X}}\ \left(\mathrm{t}\right)={\mathrm{K}}_{\mathrm{X}}^{\mathrm{drug}}(t){\Psi}_{\mathrm{drug}\_\mathrm{out}}^{(4)}\ \left(\mathrm{t}\right) $$

The quantities ${\mathrm{K}}_{\mathrm{X}}^{\mathrm{drug}}$ are called toxicity coefficients in the following.

### Assumption 4 (first cycle effect)

The term “first cycle effect” refers to increased toxicity of chemotherapeutic drugs when applied for the first time. Accordingly, we assumed a ‘first cycle effect’ by multiplying the toxicity of the first chemotherapy cycle by a factor *f*_*c*_ ≥ 1. Hence,

$$ {\mathrm{K}}_{\mathrm{X}}^{\mathrm{drug}}(t)=\left\{\begin{array}{c}\hfill {f}_{fc}^{drug}{\mathrm{K}}_{\mathrm{X}}^{\mathrm{drug}}\hfill \\ {}\hfill {\mathrm{K}}_{\mathrm{X}}^{\mathrm{drug}}\hfill \end{array}\begin{array}{c}\hfill \kern1em \mathrm{if}\kern0.5em t<{t}_2^{\mathrm{drug}}\hfill \\ {}\hfill \mathrm{else}\hfill \end{array}\right. $$

### Assumption 5 (no interactions between drugs)

**Figure 2:**
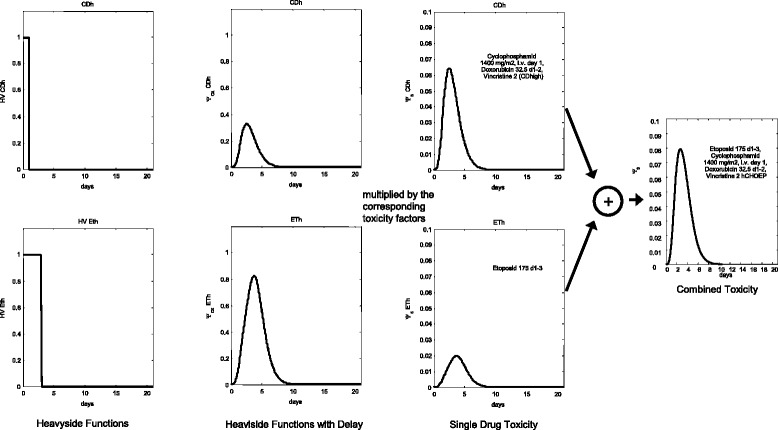
**Construction of toxicity functions exemplified by the high-CHOEP regimen and its toxic effect on stem cells.** The Heavyside functions for a single application of the combination of Cyclophosphamide, Doxorubicin and Vincristin (at time point 0) and for single Etoposide (applications at time points 0, 1 and 2) are determined (first column), delayed (second column) and multiplied with the corresponding stem cell toxicity factors (third column). Finally, the functions were added resulting in the overall toxicity function.

Most chemotherapy regimens consist of multiple drugs administered simultaneously. If toxicity functions of single drugs are available, the resulting total toxicity is obtained by adding these functions. Thus, in general, no interactions between single drugs are assumed:

$$ {\Psi}_{\mathrm{total}}^{\mathrm{X}}\ \left(\mathrm{t}\right)={\displaystyle \sum_{\mathrm{drug}}{\Psi}_{\mathrm{drug}}^{\mathrm{X}}} $$

where “drug” summarizes all drugs applied in combination. The overall process of defining toxicity functions is sketched in Figure 2.

### Assumption 6 (cell loss)

Cytotoxic drugs cause a depletion of bone marrow cells. The loss rate is proportional to the number of cells in each compartment (first order kinetics). The overall toxicity function ${\Psi}_{\mathrm{total}}^{\mathrm{X}}(t)$ defined above serves as proportionality factor, i.e.:

$$ \frac{d{C}_X(t)}{dt}={C}_X^{in}(t)\ast A(t)-\frac{C_X(t)}{T(t)}-{\Psi}_{\mathrm{total}}^{\mathrm{X}}(t){C}_X(t) $$

where *C*_*X*_ ist the content of compartment *X*.

### Assumption 7 (risk groups)

Risk groups of patients with differing toxic response can be described by different sets of corresponding toxicity parameters. Motivated by the observation that G-CSF response does not differ between elderly and younger patients [28], we assumed the same cell kinetic parameters among risk groups.

### Assumption 8 (lymphopoiesis)

Depletion of lymphocytes is modelled by the following simple equation:

$$ {C}_{WBC}(t)={C}_{ANC}(t)+{C}_{LY}\ast {e}^{-{\Psi}_{LY}(t)} $$

*C*_*WBC*_, *C*_*ANC*_ and *C*_*LY*_ are the concentrations of white blood cells, neutrophils and lymphocytes in peripheral blood. The factor ${e}^{-{\Psi}_{LY}(t)}$ quantifies the overall cytoreductive effect of chemotherapy on lymphocytes. Ψ_*LY*_ (*t*) is analogously defined as toxicities regarding granulopoiesis. Note that lymphocyte dynamics are not explicitly modelled except for this chemotherapy effect. Thus, *C*_*LY*_ = 3000/*μl* is constant, i.e. the normal concentration of lymphocytes.

### Assumption 9 (Prednisone)

Prednisone is a chemotherapeutic drug without cytotoxic effect. It is often applied for the treatment of malignant lymphoma (e.g., CHOP, CHOEP, highCHOEP and BEACOPP, see Table 1, “Summary of modelled chemotherapies”). It is well known that prednisone temporarily increases granulocyte counts caused by temporarily prolonged half-life [29-31].

**Table 1:** **Summary of modelled chemotherapies: we present names of therapies and corresponding dosage of drugs**

**Drug**	**CHOP**	**CHOEP**	**High-CHOEP**	**BEACOPP**	**BEACOPP esc.**	**TA**	**ETC**	**EC-T**	**ESHAP**	**Carboplatin + Paclitaxel**
Bleomycin (mg/m^2^)				10	10					
Carboplatin										variable
Cisplatin (mg/m^2^)									25	
Cyclophosphamid (mg/m^2^)	750	750	1400	650	1250		2500	600		
Cytarabin (mg/m^2^)									2000	
Docetaxel (mg/m^2^)						75				
Doxorubicin (mg/m^2^)	50	50	32.5	25	35	60				
Etoposide (mg/m^2^)		100	175	100	200				40	
Epirubicin (mg/m^2^)							150	90		
Methyl-prednisolon (mg)									500	
Paclitaxel (mg/m^2^)							225	175		225
Prednison (mg)	100	100	100	100	100					
Procarbacine (mg/m^2^)				100	100					
Vincristine (mg)	2	2	2	2	2					

### Assumption 10 (Cell kinetic parameters are un-affected by chemotherapy)

Besides the above mentioned (toxic) effects of chemotherapy, it is assumed that all cell-kinetic parameters of our granulopoiesis model remain unchanged. Specifically, we do not assume irreversible deterioration of granulopoiesis or reduced G-CSF response of cell stages during or after chemotherapy.

### Clinical data

Since the toxic effects of chemotherapies on bone marrow cannot directly be observed, at least one clinical dataset of patients under therapy is required to estimate it. Different schedules of the chemotherapy and different schedules of supportive G-CSF treatment provide additional information which can be used to improve toxicity estimates or to validate model predictions. Data of G-CSF application into healthy volunteers were already used to validate our pharmacokinetic and –dynamic model of G-CSF [25].

Data of 10 different chemotherapies used to treat Hodgkin’s lymphoma (HD), non-Hodgkin’s disease (NHL), breast cancer (BC) and non-small cell lung cancer (NSCLC) are available (Table 1), either from literature or from cooperating clinical study groups (German Hodgkin’s Lymphoma Study Group (Professor Engert), German High-Grade Non-Hodgkin’s Lymphoma Study Group (Professor Pfreundschuh), German Breast Group (Professor von Minckwitz)). Considering different cytotoxic drug and G-CSF schedules, data of 33 different chemotherapies are available (Table 2).

**Table 2:** **Clinical data sets used for modelling: we present disease, chemotherapy protocol and corresponding G-CSF schedules**

	**G-CSF derivative**	**Administration**	**Disease**	**Chemotherapy**	**Reference**
01	Filgrastim	5 μg/kg, days 2-13	Breast cancer	TA	[32]
02	Filgrastim	5 μg/kg, days 2-6	NSCLC	CP	[33]
03	Filgrastim	480 μg/kg, days 6-13	NHL	high-CHOEP-14*	[34]
04	Pegfilgrastim	30 μg/kg, day 2	Breast cancer	TA	[32]
05	Pegfilgrastim	60 μg/kg, day 2	Breast cancer	TA	[32]
06	Pegfilgrastim	100 μg/kg, day 2	Breast cancer	TA	[32]
07	Pegfilgrastim	6000 μg/kg, day 2	Breast cancer	TA	[35]
08	Pegfilgrastim	6000 μg/kg, day 2	Breast cancer	TA	[36]
09	Pegfilgrastim	30 μg/kg, day 2	NSCLC	CP	[33]
10	Pegfilgrastim	100 μg/kg, day 2	NSCLC	CP	[33]
11	Pegfilgrastim	300 μg/kg, day 2	NSCLC	CP	[33]
12	Pegfilgrastim	6000 μg/kg, day 2	NHL	CHOP-14	[37]
13	Pegfilgrastim	6000 μg/kg, day 2	DLBCL	R CHOP-14	[38]
14	-	-	NHL	CHOP-21* (young)	[39]
15	-	-	NHL	CHOP-21* (elderly)	[40]
16	-	-	NHL	CHOEP-21* (young)	[39]
17	-	-	NHL	CHOEP-21* (elderly)	[40]
18	-	-	HD	BEACOPP-21*	[41]
19	-	-	Breast cancer	EC-T*	[42]
20	Filgrastim	480 μg/kg, days 4-13	NHL	CHOP-14* (young)	[39]
21	Filgrastim	480 μg/kg, days 4-13	NHL	CHOP-14* (elderly)	[40]
22	Filgrastim	480 μg/kg, days 6-12	NHL	CHOP-14*	[43]
23	Filgrastim	480 μg/kg, days 4-13	NHL	CHOEP-14* (young)	[39]
24	Filgrastim	480 μg/kg, days 4-13	NHL	CHOEP-14* (elderly)	[40]
25	Filgrastim	480 μg/kg, days 6-13	NHL	high-CHOEP-21*	[43]
26	Filgrastim	480 μg/kg, days 8-13	HD	BEACOPP-14*	[44]
27	Filgrastim	480 μg/kg, days 8-15	HD	BEACOPP-21 escalated*	[41]
28	Filgrastim	480 μg/kg, days 3-10	Breast cancer	E-T-C*	[42]
29	Filgrastim	5 μg/kg, days 5-16	relapsed or persistent HD or NHL	ESHAP	[45]
30	Pegfilgrastim	6000 μg/kg, day 2	NHL	CHOP-14*	[46]
31	Pegfilgrastim	6000 μg/kg, day 4	NHL	CHOP-14*	[46]
32	Pegfilgrastim	100 μg/kg, day 6	relapsed or persistent HD or NHL	ESHAP	[45]
33	Pegfilgrastim	6000 μg/kg, day 2	DLBCL	R CHOP-14	[47]

Scenarios with access to raw data are denoted with*.

Data sets comprise time series data of G-CSF serum concentrations, ANC or WBC of patients under therapy. For modelling issues, we used patient’s medians throughout.

### Parameter estimation

Pharmakokinetic and pharmakodynamic parameters for Filgrastim and Pegfilgrastim are described elsewhere and remained unchanged in the present work [25]. In our model, the toxicity of a chemotherapy regimen is characterised by a set of cell-stage and drug specific toxicity parameters (for S, CG, PGB, MGB, LY) and a drug specific delay parameter. Parameter estimation was realised using an algorithm based on evolutionary strategies. Evolutionary strategies are stochastic algorithms used for numerical optimization [48]. The cost function to calculate model fitness was defined as:

$$ {\displaystyle {\int}_{{}_{t_0}}^{{}_{t_1}}\Big| \log {f}_{\mathrm{model}}}\left(t,\mathrm{k}\right)- \log {f}_{\mathrm{data}}(t)\Big|dt\to \min, $$

where *t*_0_ is the time of the first data point, *t*_1_ is the last data point, *f*_model_ (*t*, k) is the solution of the model equation system for the granulocyte compartment at the time of *t* (*t*_0_ ≤ *t* ≤ *t*_1_) based on the parameter set *k* = {*k*_1_, …, *k*_*n*_} and *f*_data_ (*t*) is the linearly interpolated data curve. Agreement of logarithms was pursued since cell counts are usually log-normally distributed.

We split the data of the NHL trial (CHOP-like chemotherapies of high-grade non-Hodgkin’s disease) into young and elderly patients to account for risk specific toxicities (chemotherapy assumption 7, see above). The toxicity parameters were estimated in a stepwise manner starting with simple chemotherapies which require only a few parameters estimates. More complex chemotherapies were modelled by estimating toxicity parameter sets for yet unconsidered drugs or drug combinations. Toxicity parameters estimated in earlier fitting steps were kept constant throughout the fitting process. The parameters for the first cycle effect and the two delay parameters are kept constant for different dose levels and for young and elderly patients as well. If drugs are always applied in combinations, it is impossible to separate the toxic effects of its components. In these cases, a single set of toxicity parameters was estimated for the combination.

In more detail:

1. We estimated CHOP parameters separately for elderly patients (scenarios 13, 15, 21, 30, 31 of Table 2, chemotherapy dosings can be found in Table 1), and young patients (scenarios 14, 20, 33). We assumed higher toxicity for elderly patients [49].
2. Using CHOEP data sets and the parameters found in step 1, we determined parameter settings for Etoposide 100 mg/m^2^ for young (scenarios 16, 23) and elderly patients (scenarios 17, 24).
3. Using BEACOPP basis data sets 18 and 26 and the parameters for Etoposide 100 for young patients estimated in step 2 we determined parameter settings for the combination of Cyclophosphamide 650 mg/m^2^, Doxorubicin 25 mg/m^2^ and Vincristine 2 mg, with the constraint that the parameter values of this combination must be smaller than those for CHOP young (because of lower or equal dosage of Cyclophosphamide, Doxorubicine and Vincristine compared to CHOP). Parameters for Bleomycin 10 mg/m^2^ and Procarbacine 100 mg/m^2^ were also determined.
4. With the data set 27 (BEACOPP escalated) the parameter settings for the combination of Cyclophosphamide 1250 mg/m^2^, Doxorubicin 35 mg/m^2^ and Vincristine 2 mg, and for Etoposide 200 mg/m^2^ were estimated with the constraint that the parameter values must be larger than those estimated for BEACOPP basis.
5. Taking the high-CHOEP data sets 3 and 25, we estimated parameters for the combination Cyclophosphamide 1400 mg/m^2^, Doxorubicin 32.5 mg/m^2^ and Vincristine 2 mg, and for Etoposide 175 mg/m^2^ with the constraint that parameter values must be larger than for CHOEP young.
6. Independently of the previous settings, parameters are determined for Doxorubicin 60 mg/m^2^ and Docetaxel 75 mg/m^2^ using data sets 1, 4–8.
7. Using data sets 2, 9–11, parameters are determined for the combination of Carboplatin and Paclitaxel 225 mg/m^2^.
8. Using simultaneously the data sets of E-T-C (data set 28) and EC-T (data set 19), the parameter settings for Epirubicin (dose 90 mg/m^2^ or 150 mg/m^2^), Paclitaxel (dose 175 mg/m^2^ or 225 mg/m^2^) and Cyclophosphamide (dose 600 mg/m^2^ or 2500 mg/m^2^) were determined with the constraint that lower doses have lower values of toxicity parameters.
9. With the ESHAP data set 29, parameter settings for Etoposide 40 mg/m^2^, Cytarabine 2000 mg/m^2^ and Cisplatin 25 mg/m^2^ were determined.

Three scenarios were not used for parameter estimation and served as model validation: WBC data from non Hodgkin lymphoma patients treated with CHOP-14 and Filgrastim on day 6–12 (data set 22), ANC data of patients with relapsed or persistent HD or NHL, treated with ESHAP and Pegfilgrastim 100 μg/kg on day 6 (data set 32) and WBC and G-CSF serum level data from non Hodgkin lymphoma patients treatet with CHOP-14 and Pegfilgrastim 6000 μg on day 2 (data set 12).

### Quantification of myelotoxicity

In order to compare toxicity of different chemotherapy and G-CSF scenarios, it is necessary to quantify the degree of reduction of granulocytes during the course of the therapy. There is evidence that the risk for infectious complications in neutropenic patients depends on the depth as well as on the duration of neutropenia [50,51]. Therefore, we defined the area between a certain threshold and the model curve below the threshold (AOC) as an appropriate summary measure for neutropenia/leukopenia or severity of reduction of other bone marrow cell stages. We used 2.000/μl and 4.000/μl as thresholds for total cell counts of neutrophils and leukocytes respectively. For normalized cell counts we always use the steady-state value 1 as threshold. The AOC was either used to compare overall toxicity between schedules or served as a target measure for optimizing G-CSF schedules.

### Technical implementation

The model equations were programmed and solved on a standard personal computer using the numeric computation software Matlab 7.5.0.342 (R2007b) and the integrated Simulink toolbox v7.0 (The MathWorks, Natick, MA). Model simulations were performed by numerical integration of the ODE system. For our model, evaluation of functions is expensive. Therefore, we used the variable step solver from Adams and Bashford (ode113).

## Results

Applying our model, we simulated 14 different chemotherapy scenarios (TA, CP, CHOP-14, CHOP-21, CHOEP-14, CHOEP-21, high CHOEP-14, high CHOEP-21, BEACOPP-14, BEACOPP-21, BEACOPP escalated, EC-T, E-T-C, and ESHAP) including 14 different drugs. Taking into account individual risk groups, we estimated a total of 12 different parameter sets. Considering different schedules of Pegfilgrastim and Filgrastim, 33 scenarios were modelled. First, we study the qualitative behaviour of our resulting chemotherapy model.

### Qualitative behaviour of the chemotherapy model

**Figure 3:**
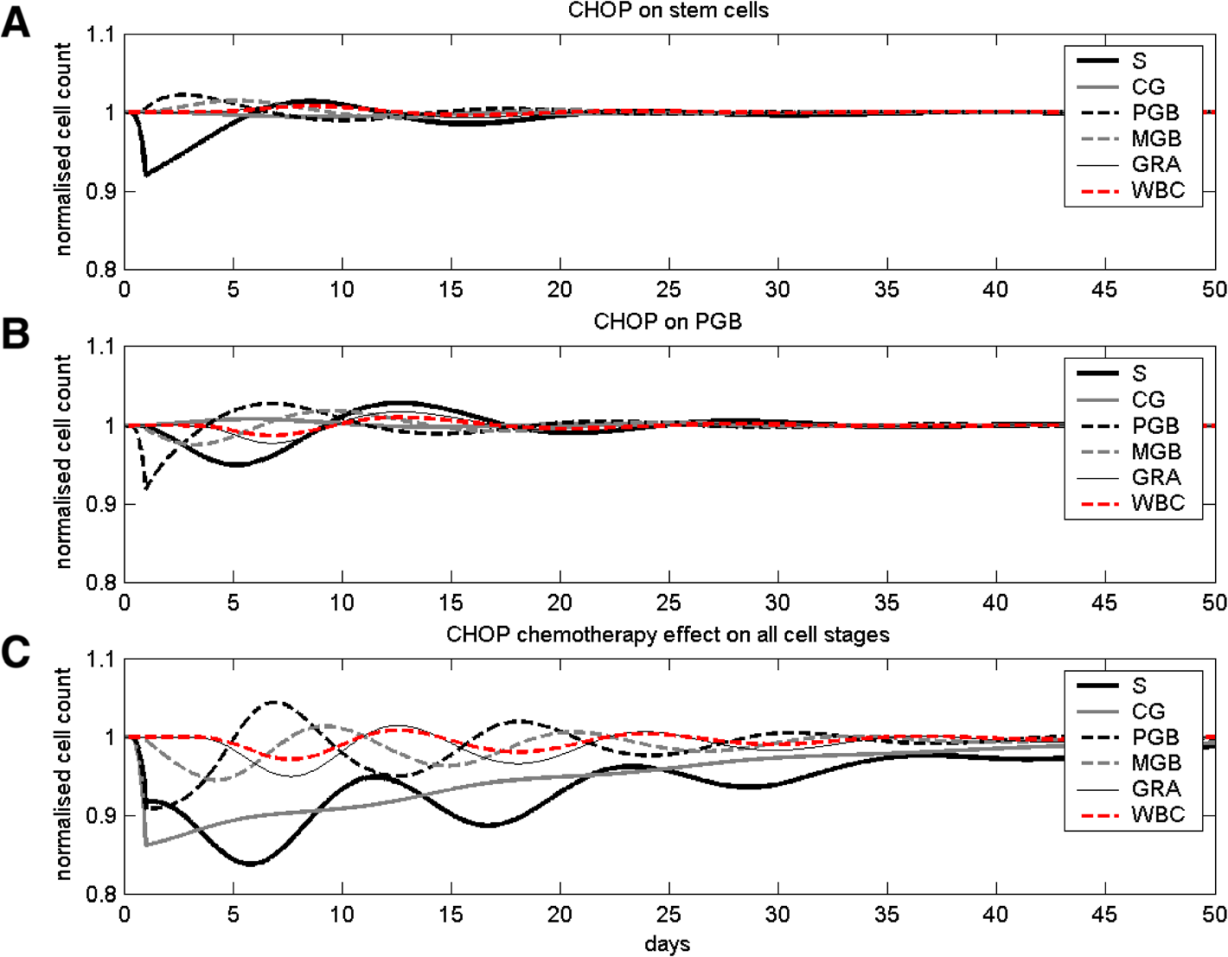
**Toxicity of CHOP chemotherapy.** Time courses of normalised cell counts of different cell stages after a single application of CHOP chemotherapy. **A:** CHOP effect only on stem cells. **B:** CHOP effect only on PGB. **C:** CHOP effect on all cell stages.

In Figure 3, we studied the behaviour of our chemotherapy model on the basis of simplified chemotherapy actions. The estimated parameter set for CHOP in elderly patients was considered for this purpose. At first, the effect of an isolated stem cell kill imposed by the CHOP chemotherapy is simulated (Figure 3A). As a result, the stem cells are diminished quickly, while the later cell stages decrease with some delay. After mild oscillations, the cell counts approach normal levels.

Next, we simulated an isolated CHOP chemotherapy effect on the compartment PGB alone (Figure 3B). PGB decrease immediately, and, after certain delay, other cell stages are reduced too. After oscillation, the cell counts return to normal levels similar to the isolated stem cell kill.

In Figure 3C we show the results of CHOP toxicity affecting all cell stages. This toxic effect is equivalent to later simulations of clinical scenarios involving CHOP. The figure shows that due to the combined toxicity on all cell stages, the compartment CG is most seriously affected. Recovery of the system takes much more time than in the above mentioned scenarios.

### Simulations of simple chemotherapies

**Figure 4:**
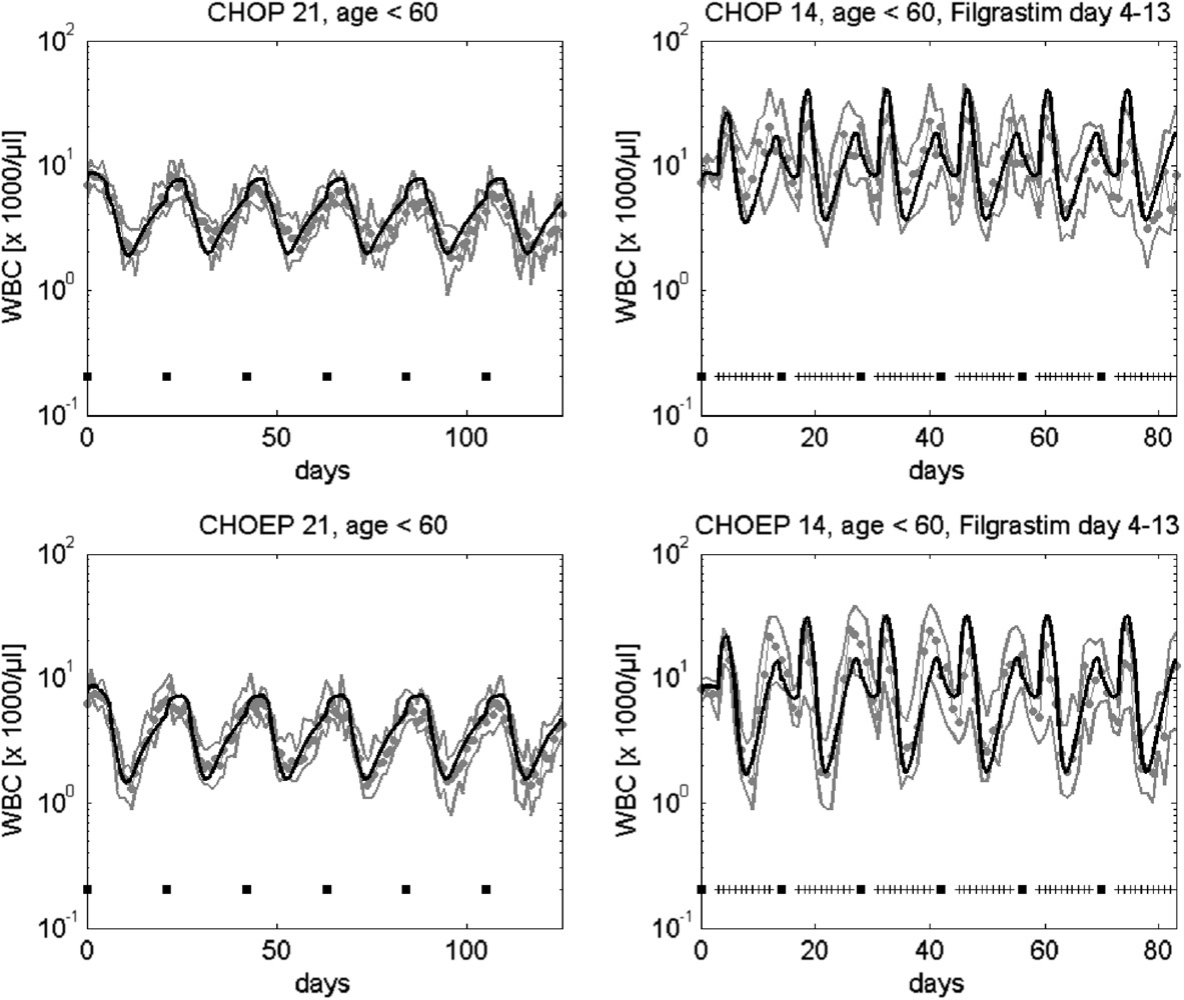
**Simulation results for CHOP-21, CHOP-14, CHOEP-21, CHOEP-14, younger patients.** We show simulated cell counts for CHOP and CHOEP chemotherapy. Dots represent patient medians, grey lines represent interquartile range of patient data, black squares correspond to chemotherapy administrations, “+” correspond to days with G-CSF-injections. Clinical data originate from our collaborating clinical trials group [39], see Table 2.

According to step 1 of our estimation procedure, we fitted parameters of simple chemotherapy scenarios first. Simple chemotherapies refer to those comprising either a small number of different cytotoxic drugs or drug combinations applied at the same time. This applies for data sets 1–12, 13–17, 20, 21, 23–25, 30, 31 and 33, where only one or two toxicity parameters per cell stage are required to describe the therapy. As example, a comparison of model and data for the CHOP and CHOEP young scenarios with and without G-CSF treatment can be found in Figure 4. Other scenarios can be found in the appendix, Additional file 1: Figures A3-A8. Estimated parameter sets (Table 3) resulted in a good agreement of model and data.

**Table 3:** **Toxicity parameters: Each drug or drug-combination is characterized by a set of eight parameters**

		**FC (txFC)**	**Delay (Deltx)**	**S (txS)**	**CG (txCG)**	**PGB (txPGB)**	**MGB (txMGB)**	**Delay WBC (Deltx…wbc)**	**tox WBC (txWBC)**
Cyclophosphamid 750 mg/m^2^ d1, Doxorubicin 50 mg/m^2^ d1, Vincristine 2 mg d1 (CHOPo)	CHOP, age > 60	1.11E + 00	6.35E-02	2.16E-01	3.70E-01	2.34E-01	1.69E-04	1.37E-02	1.27E + 01
Etoposide 100 mg/m^2^/d d1-3 (ETo)	CHOEP, age > 60	1.10E + 00	6.84E-02	2.69E-04	1.97E-02	1.04E + 00	3.00E-06	3.50E-02	7.49E-01
Cyclophosphamid 750 mg/m^2^ d1, Doxorubicin 50 mg/m^2^ d1, Vincristine 2 mg d1 (CHOPy)	CHOP, age < 60	1.11E + 00	6.35E-02	1.94E-01	3.70E-01	1.06E-01	1.30E-04	1.37E-02	1.08E + 01
Etoposide 100 mg/m^2^/d d1-3 (ETy)	BEACOPP, CHOEP, age < 60	1.10E + 00	6.84E-02	1.91E-04	5.46E-03	4.02E-01	2.00E-06	3.50E-02	9.30E-02
Procarbazine 100 mg/m^2^/d, d1-7 (PROC)	BEACOPP (escalated)	1.09E + 00	1.30E-02	2.42E-03	1.03E-02	5.19E-02	7.80E-05	1.00E-05	1.00E-05
Cyclophosphamid 650 d1, Doxorubicin 25 mg/m^2^ d1, Vincristine 2 mg d1 (CD)	BEACOPP	1.11E + 00	6.35E-02	6.47E-04	3.70E-01	1.48E-02	4.10E-05	1.37E-02	1.08E + 01
Bleomycin 10 mg/m^2^ (VB)	BEACOPP (escalated)	1.32E + 00	3.33E-03	1.20E-02	3.01E-02	1.57E-02	6.00E-06		
Cyclophosphamid 1250 mg/m^2^ d1, Doxorubicin 35 mg/m^2^ d1, Vincristine 2 mg d1 (CDesk)	BEACOPP escalated	1.11E + 00	6.35E-02	2.12E-01	3.70E-01	2.27E-01	1.84E-04	1.37E-02	1.09E + 01
Etoposide 200 mg/m^2^/d d1-3 (ETesk)	BEACOPP escalated	1.10E + 00	6.84E-02	1.91E-04	1.18E-02	4.02E-01	3.00E-06	3.50E-02	2.67E + 01
Cyclophosphamid 1400 mg/m^2^, i.v. day 1, Doxorubicin 32.5 mg/m^2^/d d1-2, Vincristine 2 mg d1 (CDh)	high CHOEP	1.11E + 00	6.35E-02	1.94E-01	7.26E-01	3.37E-01	1.77E-04	1.37E-02	1.10E + 01
Etoposide 175 mg/m^2^/d d1-3 (ETh)	high CHOEP	1.10E + 00	6.84E-02	2.42E-02	4.87E-02	6.41E-01	8.00E-06	3.50E-02	7.29E + 00
Carboplatin, Paclitaxel 225 mg/m^2^ (CP)	Carboplatin, Paclitaxel	1.00E + 00	7.71E-02	5.05E-04	6.00E + 01	6.00E + 01	2.89E-04		
Doxorubicin 60 mg/m^2^, Docetaxel 75 mg/m^2^ (TA)	Doxorubicin, Docetaxel	2.01E + 00	7.24E-02	1.39E-02	2.28E-01	4.22E + 00	3.20E-05		
Paclitaxel 225 mg/m^2^, 3-h-Infusion (Pacli or P225)	ETC	1.05E + 00	1.73E-02	2.46E-01	8.21E-01	3.50E + 00	7.07E-04	1.97E-01	2.65E-01
Paclitaxel 175 mg/m^2^ 3-h-Infusion (Pacli or P175)	EC-T	1.05E + 00	1.73E-02	7.00E-05	8.21E-01	3.50E + 00	7.01E-04	1.97E-01	2.65E-01
Cyclophosphamid 600 mg/m^2^, 24-h-Infusion (Cyclo or C600)	EC-T	1.01E + 00	6.40E-02	1.99E-01	7.43E-01	1.15E-01	1.40E-04	2.50E-02	8.99E + 00
Cyclophosphamid 2500 mg/m^2^, 24-h-Infusion (Cyclo or C2500)	ETC	1.01E + 00	6.40E-02	1.99E-01	7.58E-01	1.27E-01	1.43E-04	2.50E-02	1.31E + 01
Epirubicin 90 mg/m^2^ 3-h-Infusion (Epi or E90)	EC-T	1.99E + 00	4.48E-02	1.80E-05	5.51E-02	1.80E-01	5.18E-04	2.42E-02	3.96E + 00
Epirubicin 150 mg/m^2^ 3-h-Infusion (Epi or E150)	ETC	1.99E + 00	4.48E-02	2.53E-03	1.56E + 00	6.62E + 00	1.58E-02	2.42E-02	3.01E + 01
Cytarabine 2000 mg/m^2^ (Cyta)	ESHAP	1.00E + 00	1.31E-01	4.64E-01	8.80E-03	8.84E-03	2.32E-03		
Cisplatin 25 mg/m^2^/d (Cisp)	ESHAP	1.00E + 00	6.32E-02	3.24E-02	1.93E-01	9.60E-03	4.10E-04		
Etoposide 40 mg/m^2^ (ET40)	ESHAP	1.10E + 00	6.84E-02	0.00E + 00	1.62E-02	8.65E-02	2.00E-06		

Parameters also depend on drug doses and age (≤60 vs. >60 years).

### Chemotherapy model: more complex chemotherapy simulations

**Figure 5:**
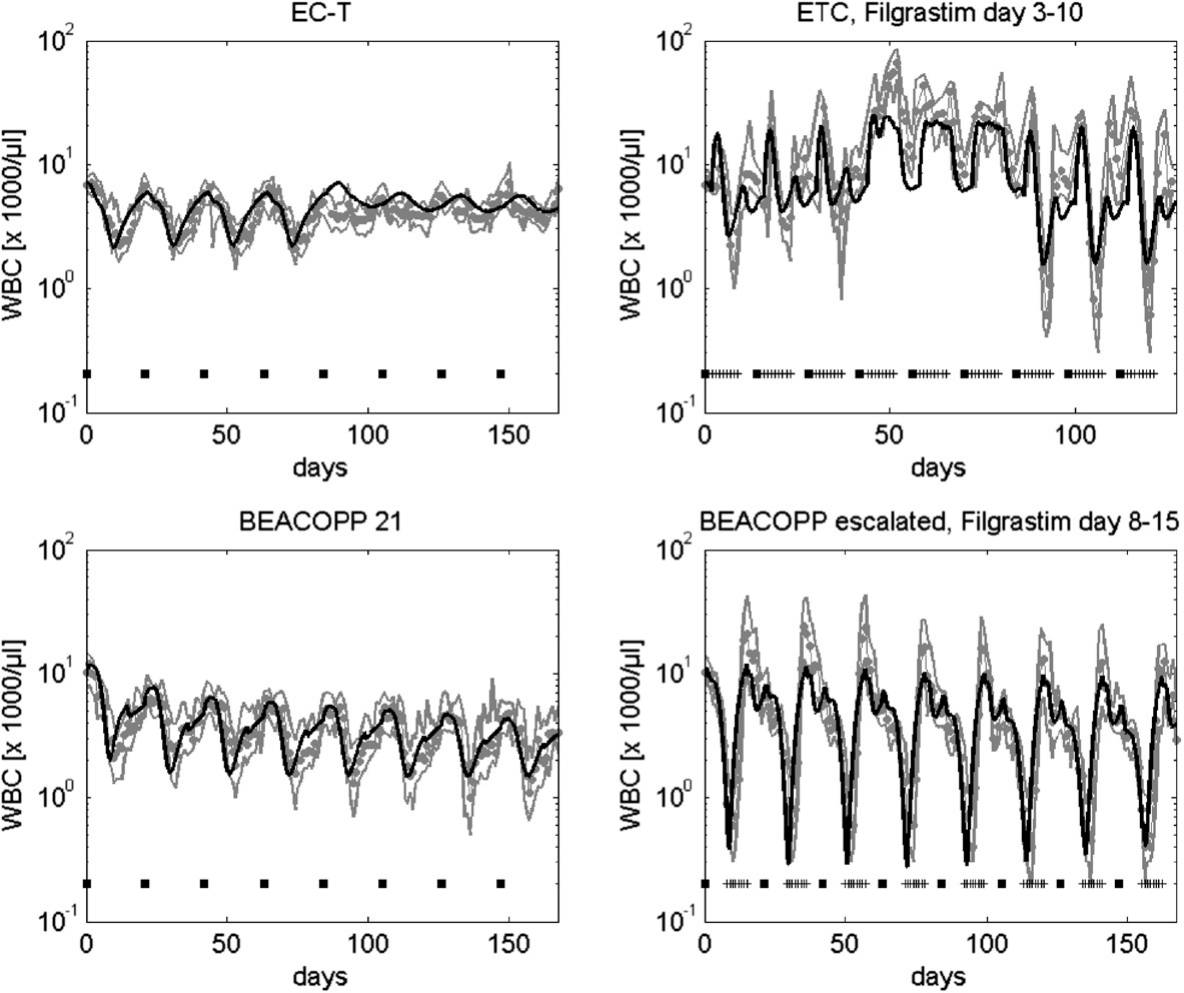
**Simulation results of selected complex chemotherapies.** We present results for the two breast cancer therapies, EC-T and ETC with Filgrastim on days 3–10 (first row). Note that in these schedules, chemotherapeutic drugs differ between cycles: For EC-T the drugs epirubicine and cyclophosphamide where applied in combination in the first four cycles. The single drug paclitaxel was applied for the last four cycles. For ETC, the single drug epirubicine was applied in three cycles followed by three cycles of paclitaxel and three cycles of cyclophosphamide. We also present two therapies of advanced Hodgkin’s lymphoma, BEACOPP-21 and BEACOPP escalated with Filgrastim on days 8–15, in which multiple drugs are administered at different time points per cycle (second row). Dosages can be found in Table 1. Dots represent patient medians, grey lines represent interquartile range of patient data, squares represent chemotherapy administrations, “+” denote G-CSF-injections.

In the next step, more complex chemotherapies containing a higher number of cytotoxic drugs or more complex schedules are modeled (scenarios 18, 26–29). If applicable, toxicity estimates of drugs or drug combinations established in the previous section were kept constant. Comparisons of model and data for selected scenarios can be found in Figure 5. All other scenarios can be found in the appendix (Additional file 1: Figures A9-A10).

### Quantification of chemotherapy toxicity

As can be seen from Figures 4 and 5 and those of other scenarios presented in the appendix, our model assumptions regarding chemotherapy action and corresponding toxicity parameters resulted in a reasonable fit of almost all scenarios considered.

Our toxicity parameters can be interpreted as the strength of chemotherapy damage on the respective cell stage. We now discuss and interprete these parameters in more detail. An overview for different drugs and drug doses can be found in Table 3.

Sensitivity analysis (Additional file 1: Figures A1, A2) revealed that among bone marrow toxicities, those estimated for stem cells showed the highest precision in most scenarios. The LY toxicity and the delay parameters are sensitive too.

**Figure 6:**
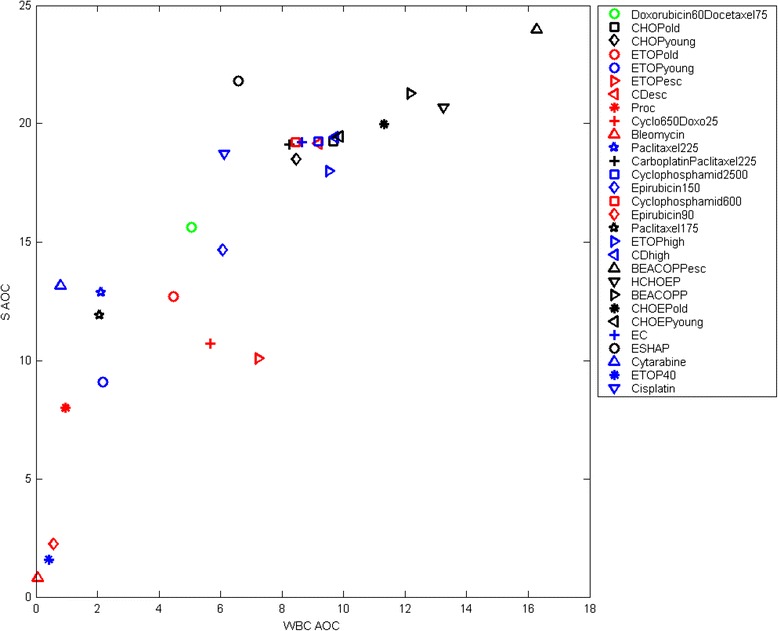
**Correlation of stem cell toxicity and peripheral toxicity.** We determined cumulative toxicities of stem cells and mature blood cells for each drug or drug combination considered. Toxicities are expressed in terms of AOC of normalized cell counts applying the steady-state value 1 as threshold. AOC is calculated over 28 days. Only a single injection of chemotherapy was simulated for this purpose. The unit of AOC is “d”. A good correlation between stem cell and peripheral toxicity can be observed (Spearman’s r = 0.88). Toxicity relations between schedules are plausible. Note that schedules may be attributed to different groups of patients.

Figure 6 shows the relation between stem cell toxicity and resulting WBC toxicity. The correlation (Spearman) is 0.88, i.e. the stem cell toxicity is a good predictor of the overall toxicity. Additional file 1: Figure A11 of the supplement material shows the correlation of MGB AOC and WBC AOC. In Additional file 1: Figure A12 the correlation of ANC AOC and WBC AOC is depicted. Both are highly correlated too (MGB AOC vs. WBC AOC r = 0.94, ANC AOC vs. WBC AOC r = 0.92).

For Cyclophosphamide (with or without Doxorubicin and Vincristin) we estimated a high stem cell toxicity in agreement with the literature (e.g. [52]). For Etoposide, we obtained a rather low stem cell toxicity, even for increased dose levels, and higher toxicity to later cell stages. This also complies with the literature (e.g. [2]). Due to the rather small haematotoxic influence of Vincristine, we abstained from determining a separate parameter set for Vincristine [52].

Two anthracyclines were considered, Doxorubicine and Epirubicine. The first one was always applied in combination with other drugs, namely with Cyclophsphamide for therapies of lymphoma diseases and with Docetaxel for the TA regimen as adjuvant breast cancer therapy. Therefore, no separate parameter set of Doxorubicine alone could be derived. In contrast, Epirubicine was applied as single drug in the ETC therapy of breast cancer patients. This allows us to derive a separate set of toxicity parameters for two dose levels of Epirubicine (90 and 150 mg/m^2^) showing a considerable, dose-dependend stem cell toxicity in agreement with the literature [52].

According to our assumption 5, cytotoxic drugs are assumed to contribute to overall toxicity independently of each other. This does not apply for the combination of Carboplatin and Paclitaxel for which it is known that the combination is less toxic than the single drugs [53]. Therefore, a new set of toxicity parameters was determined for this drug combination, which indeed resulted in lower estimates than for Paclitaxel alone (see Table 3).

We assumed that risk groups of haematotoxicity can be traced back to differences in toxicity parameters (assumption 7). This assumption worked fine if comparing the toxicity outcomes of young and elderly patients treated with CHOP or CHOEP chemotherapies. For both risk groups the agreement of model and data is fine while corresponding toxicity parameters are higher in elderly patients.

### Validation

**Figure 7:**
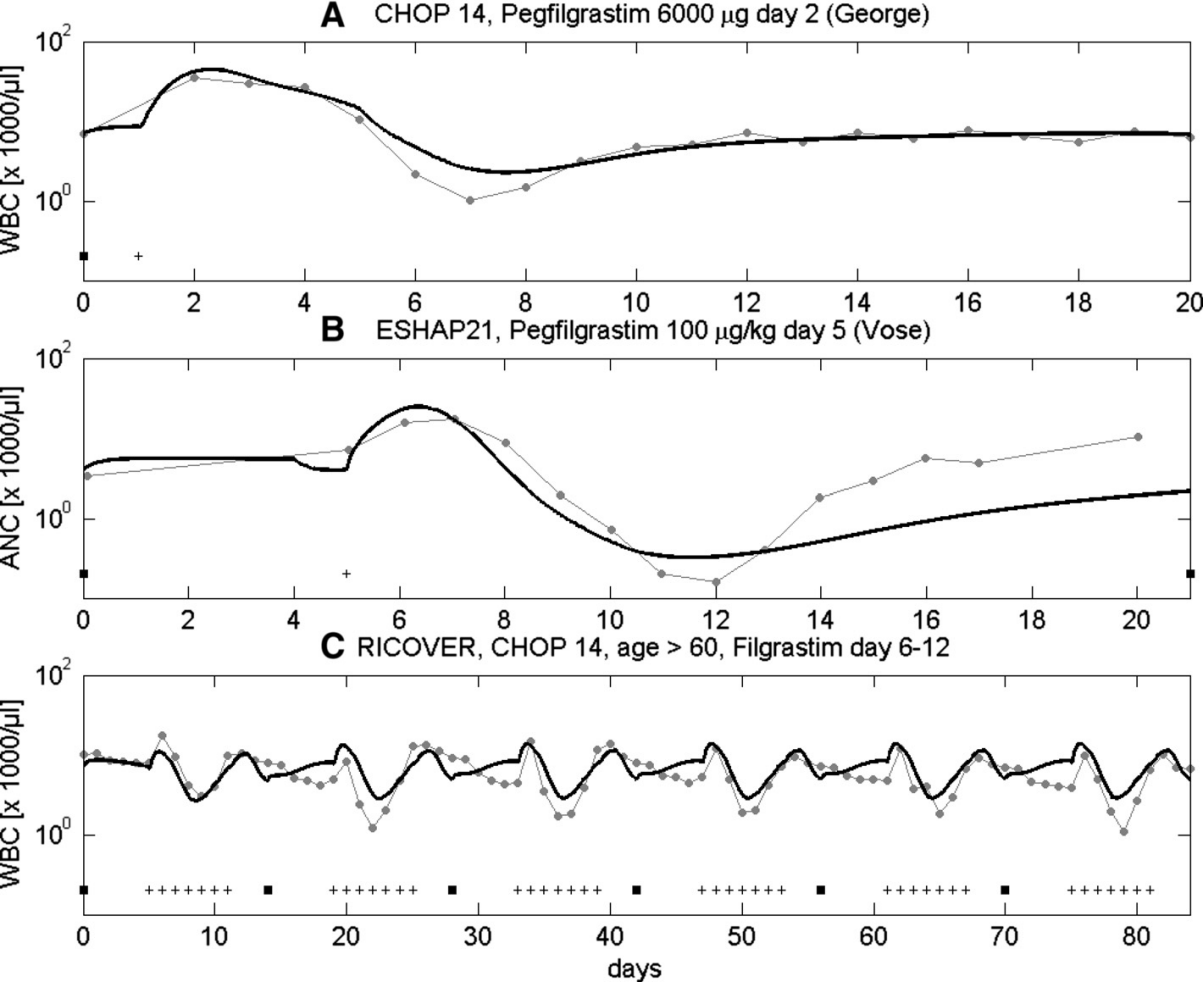
**Validation scenarios.** We compare model results with clinical data from validation scenarios: **A**: CHOP with Pegfilgrastim 6000 μg on day 2 (data: [37]), **B**: ESHAP with Pegfilgrastim on day 5 (data: [45]), **C**: CHOP with 480 μg Filgrastim on cycle-days 6–12, for elderly patients, data: [43]. Dots represent patient medians, squares represent the chemotherapy administrations, + are time points with G-CSF-injections.

Data sets not used for parameter fitting served as validation scenarios of our model. This requires that the corresponding chemotherapy parameters were determined on the basis of other data sets. Scenarios 12, 22, 32 fulfill these requirements. Figure 7 shows the agreement of model and data for validation scenarios.

### Model predictions

**Figure 8:**
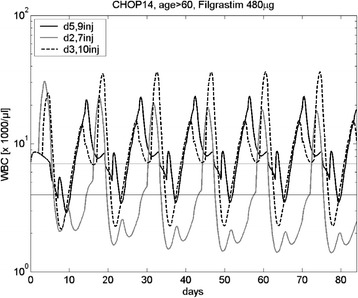
**Analysis of CHOP with Filgrastim (age > 60 years).** Simulated cell counts for CHOP-14, with Filgrastim 480 μg on days 5–13 (black line), days 2–8 (grey line) and days 3–12. Filgrastim at day 2–8 results in particularly low leukocyte counts. Better results are obtained by the Filgrastim application on days 3–12 or days 5–13.

A key feature of our model is that it allows simulations of alternative G-CSF schedules and its effects on overall leukotoxicity. We demonstrate this on the basis of the CHOP regimen for elderly patients: Using toxicity parameters estimated for CHOP based on the G-CSF schedules presented above, we modified the starting time and the duration of Filgrastim treatment. Comparisons of schedules can be performed by calculating the AOC of the simulation results. Two examples of simulated G-CSF schedules are presented in Figure 8. The regimen day 2–8 results in clearly inferior AOC than the current standard (G-CSF at day 3–12). In contrast, for the schedule day 5–13 we predict a better AOC than the current standard even though the number of injections is reduced.

**Figure 9:**
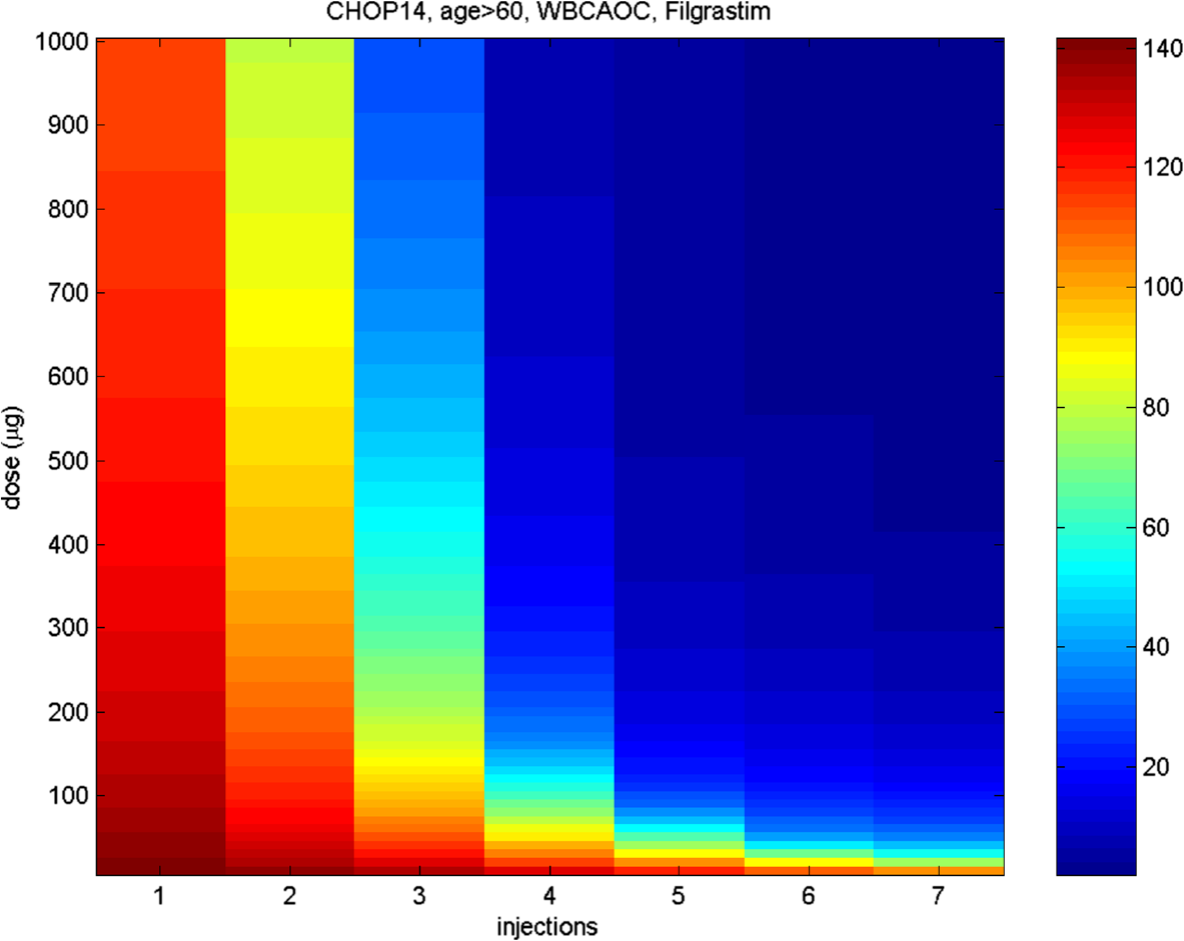
**Modified Filgrastim schedules for CHOP-14 in elderly patients.** Predicted WBC AOC (applying a threshold of 4000/μl, calculated over 84 days, unit of AOC is 1000/μl*d) under CHOP-14 with Filgrastim: G-CSF injections start at day 7 in each cycle. We modified the number of Filgrastim injections and its doses. The color scale on the right corresponds to AOC values.

We calculated the WBC AOC for 6 cycles of the CHOP-14 regimen, administered to elderly patients, in dependence on different Filgrastim doses and injection numbers starting on day seven (Figure 9). Best results are predicted, if Filgrastim injections are applied from day seven up to the end of the therapy cycle (see also [54]). Increasing G-CSF dose results only in marginal improvements.

## Discussion

Conventional cytotoxic chemotherapy plays a major role in cancer therapy. Development of intensified regimen improved the outcome of several diseases [39-41,55,56] but is limited by toxic side effects. A major, frequently dose-limiting side effect is granulotoxicity which is routinely treated with the growth factor G-CSF. A variety of pharmaceutical derivatives are available, which differ greatly in pharmacokinetic and -dynamic properties. Furthermore, outcome of growth factor treatment depends on many factors such as chemotherapy drugs used, drug doses, growth-factor derivatives and individual risk factors [57,58]. Due to this variety of variable therapy parameters, identification of optimal growth-factor schedules cannot be performed solely on the basis of clinical trials.

We recently developed a model of the pharmacokinetic and –dynamic action of the G-CSF derivatives Filgrastim and Pegfilgrastim under conventional poly-chemotherapy [25]. We also showed that the model successfully predicts the outcome of alternative G-CSF schedules [54]. However, so far only a single simple chemotherapy schedule was considered for which the data base was most comprehensive, namely the CHOP regimen used to treat high-grade non-Hodgkin’s lymphoma diseases. The major purpose of the present work is to extend the applicability of our model considering a broad range of conventional chemotherapy schedules. This requires the construction of a comprehensive model of chemotherapy action on the granulopoietic system. Making a number of biologically plausible assumptions and translating them into differential equations allowed us to predict granulocyte and leukocyte dynamics of virtually all chemotherapy scenarios with published time series data of granulocytes and leukocytes (33 scenarios comprising 10 different chemotherapies). Modelling of chemotherapies essentially requires estimation of dose, drug and cell-stage specific toxicity parameters. In consequence, our model can easily be applied to novel chemotherapy scenarios for which time series data are available allowing estimation of these parameters. We showed how the model can be used to systematically explore the outcomes of alternative G-CSF schedules for a chemotherapy for which toxicity parameters are available.

Ongoing efforts to model haematopoiesis under chemotherapy and growth-factor applications are considerable [59-84]. Most newer models consider G-CSF as the major stimulant of granulopoiesis, and account for corresponding intracellular mechanisms, as well as for receptor binding kinetics and endocytic ligand depletion [62,64,85,86]. Shochat et al. [63], and Foley et al. [59], proposed models considering both, stimulating effects of G-CSF as well as the cell depleting effects of chemotherapy. However, published models usually consider selected chemotherapy regimens. So far it has not been shown that these model concepts are valid for a broad range of chemotherapies and schedules [59,63]. First attempts to predict the performance of alternative G-CSF schedules on the basis of these models were performed [87].

In order to construct a comprehensive model of chemotherapy action on granulopoiesis, we made the following assumptions and translated them into differential equations:

### Delayed toxicity

It is assumed that the cell depleting effect of chemotherapy starts immediately after drug application. The maximum is reached after some time delay. This assumption is motivated by available time series data of murine bone marrow cellularity after a variety of cytotoxic drug applications often showing a maximum response to chemotherapy treatment at later time points even if the underlying drugs are quickly metabolized in vivo [52]. This phenomenon can be explained for example by delayed apoptosis of cells after damage, e.g. at time when cells entering their next cell cycle.

### Cell type specific toxicity

It is assumed that chemotherapy acts cell type specific which is supported by numerous experimental data [52]. This implies that toxicity parameters are assumed to be dose, drug and cell-stage specific. Most of our data sets comprise leukocyte counts instead of neutrophil profiles. To account for this fact, we accompanied our cell-kinetic model of granulopoiesis by a simple model of lymphotoxicity. This is motivated by differing dynamics of granulocytes and lymphocytes observed in chemotherapy-treated mice [88]. Further clinical evidence is provided by a trial with breast cancer patients undergoing polychemotherapy. In this study, suppressed B- and T-cell populations were still present at times when the absolute neutrophil count had returned to normal or even higher than normal values [89]. These findings are in good agreement with the results of our parameter estimation (prolonged toxicity for lymphocytes). However, to precisely quantify lymphocyte toxicity profiles, more detailed differential blood counts of patients undergoing chemotherapy would be required than currently available.

### First cycle effect

There is some evidence that the first application of chemotherapeutic drugs results in higher toxicity [81,90-92]. We modelled this effect in a phenomenologic way by multiplying toxicity parameters with a factor ≥ 1 at time of first application of chemotherapy.

### Toxicity of drug combinations

To estimate the overall toxicity of a drug combination, we achieved satisfying results by adding the toxicity parameters of single substances or groups of substances. However, there is evidence that some drug combinations interact in a paradoxical way, resulting in an overall toxicity that is smaller than the toxicity of either one of the single agents. For example, thrombocytopenia tends to be significantly less pronounced in patients treated with carboplatin when combined with paclitaxel (which by itself causes thrombocytopenia, too) [53,93,94]. The reason for the platelet sparing effect of this combination is still unknown. Therefore, it is possible that the toxicity of a drug combination cannot be simply derived by adding the toxicity parameters of their components determined in previous studies. In these cases, new parameter fittings are required.

### Action of chemotherapy on granulopoiesis

We modeled chemotherapy to act cytotoxic, rather than cytostatic. As result, cells in our model were removed from the compartments directly, whereas cell kinetic properties (amplification and transit time) are not affected. However, it is well known that many drugs act cytostatically, for example by disrupting cellular metabolism or DNA synthesis. Hence, onset and severity of chemotherapy associated myelotoxicity depend on the cell cycle. However, in our modeling framework, it is neither possible nor necessary to distinguish between cytostatic and cytotoxic effects since both result in reduced cell numbers within a relatively small time frame. Closely meshed time series data of bone marrow cell stages would be required for a more detailed modelling of this issue which however cannot be established for humans.

### Modelling risk groups

Numerous clinical risk factors regarding toxic response of patients are known such as age, sex and general health status [49]. We hypothesize that this heterogeneity can be traced back to different sets of toxicity parameters rather than cell kinetic parameters of granulopoiesis [28,95]. This assumption allowed us for example to stratify patients into risk groups described by risk-specific toxicity parameters [58]. So far, differences between younger and elderly patients could be successfully explained by higher toxicity parameters as can be seen on our toxicity parameters for etoposide and the combination of cyclophosphamide, doxorubicine and vincristine applied in CHOP-like chemotherapy regimens. For both risk groups, corresponding parameter estimates resulted in good explanation of clinical data.

Overall, our model assumptions proved to be feasible for modelling almost all published time series data after a large variety of chemotherapies and schedules.

A few scenarios, for which complete sets of toxicity parameters are available, were used to successfully validate the model. We qualitatively compared derived toxicity parameters between schedules and received clinically plausible results: We estimated for example that Bleomycin (10 mg/m^2^), Procarbacine (100 mg/m^2^) and low dose Etoposide (100 mg/m^2^) have low granulotoxicity according to clinical experiences. In contrast, BEACOPP escalated and high-CHOEP are among the most toxic therapies in agreement with high percentages of grade 3 and 4 leucopoenia observed in these patients [43,49].

We observed that stem cell toxicities of drugs or drug combinations correlate well with resulting leukocyte toxicity (r = 0.88, Figure 6) indicating that the parameter of stem cell toxicity is the most sensitive of our parameters characterizing chemotherapy toxicity. In contrast, the parameters for CG, PGB and MGB are less well characterized, in general. For these three cell stages, we have to acknowledge that higher toxicity at later stages can somewhat be compensated with lower toxicity at earlier cell stages and vice versa.

Finally, we demonstrated how the model could be used to make clinically relevant predictions regarding the outcome of alternative growth-factor schedules after chemotherapy. This requires that the toxicity parameters of the considered therapy are available. Then, the model can be used to simulate and compare alternative growth-factor schedules. Since hard clinical endpoints such as febrile neutropenia, use of antibiotics or length of stay in hospital cannot be addressed by our modelling, it was necessary to use surrogate markers in order to compare efficacy of G-CSF prophylaxis between schedules. We used the area between model curve and the line of 2.000/μl neutrophils or 4.000/μl leukocytes for this purpose. Other measures of relative toxicity are discussed elsewhere [95].

Finally, we have to acknowledge that the present model only allows median predictions while critical time-courses are clinically more relevant and therapy-limiting. Although this aspect is not yet covered, there is a clear perspective towards modelling individual data either by fitting parameter sets for patient risk groups or by assuming distributions of model parameters. Accordingly, we plan to extend our model and apply it in order to support improvement and individualisation of G-CSF therapies. Since we have the clear intention to apply our model in clinical contexts, we also plan public release of our model software in the near future.

## Conclusion

We successfully developed a bio-mathematical model of granulopiesis under chemotherapy and applications of the G-CSF derivatives Filgrastim and Pegfilgrastim. Our model is able to simulate neutrophil and leukocyte profiles in the peripheral blood under various chemotherapies, with and without Filgrastim or Pegfilgrastim. The model consistently explains available clinical data, and can be used to predict the performance of alternative G-CSF schedules.

## Additional file

Additional file 1:
**Modelling chemotherapy effects on granulopoiesis: Supplement Material.** The file GraPaper-2-Appendix.pdf contains sensitivity analysis and further simulation results.
