# Supplementary material for: Modelling chemotherapy effects on granulopoiesis
Source: BMC Syst Biol. 2014 Dec 24;8:138. doi: 10.1186/s12918-014-0138-7 (PMC4302124; doi:10.1186/s12918-014-0138-7)
Supplement: Additional file 1: — Modelling chemotherapy effects on granulopoiesis: Supplement Material. The file GraPaper-2-Appendix.pdf contains sensitivity analysis and further simulation results. [file 12918_2014_138_MOESM1_ESM.pdf]

Modelling chemotherapy effects on granulopoiesis

Supplement Material

Sibylle Schirm[1,2], Christoph Engel[1], Markus Loeffler[1], Markus Scholz[1,2]

[1]Institute for Medical Informatics, Statistics and Epidemiology (IMISE), Medical Faculty, University of Leipzig, Germany

[2] LIFE Research Center

Sensitivity Analysis

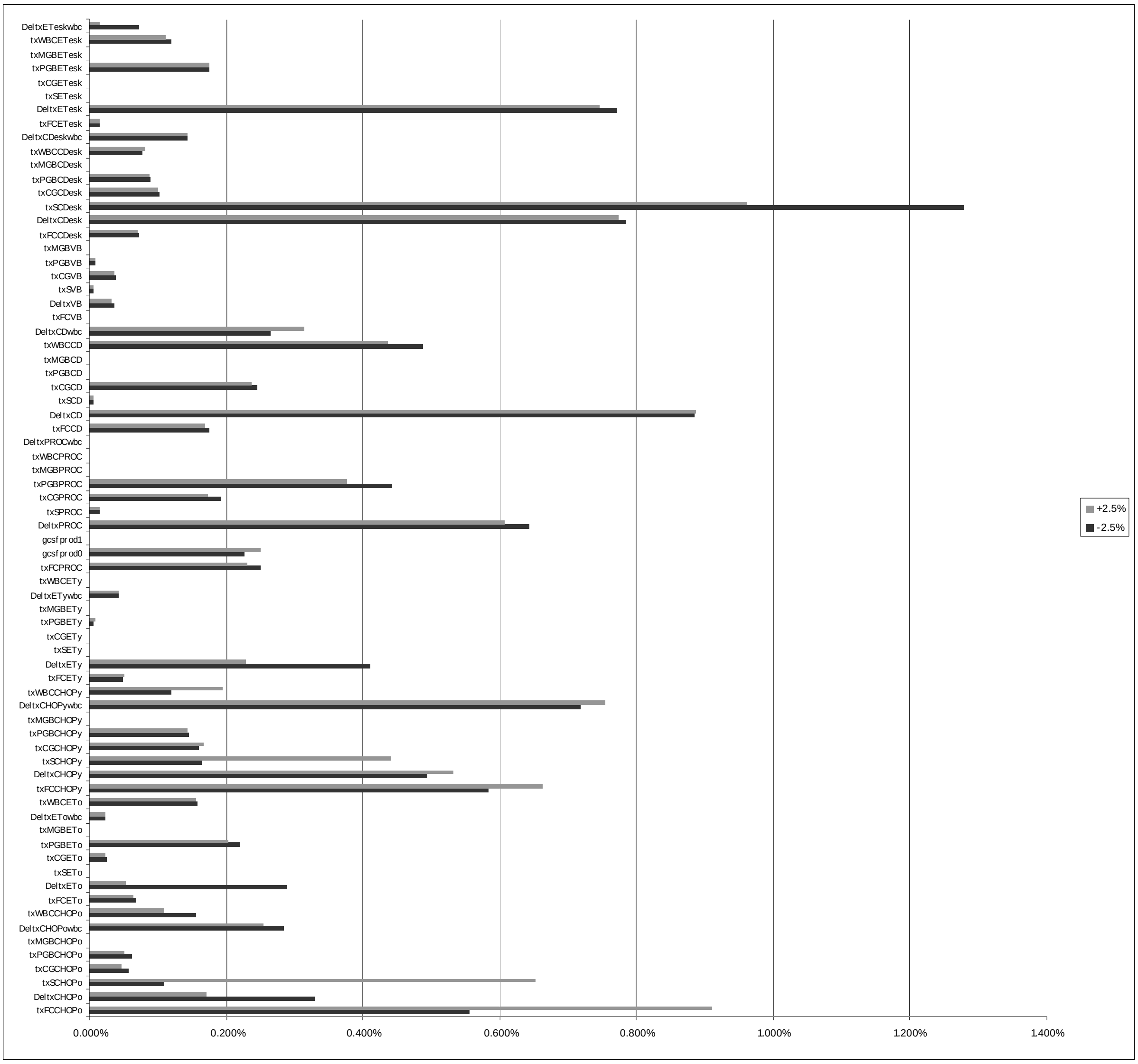

Figure A1: Sensitivity of chemotherapy parameters (part 1). The diagram depicts the change in the fitness value (%) after a parameter modification of 2.5%.

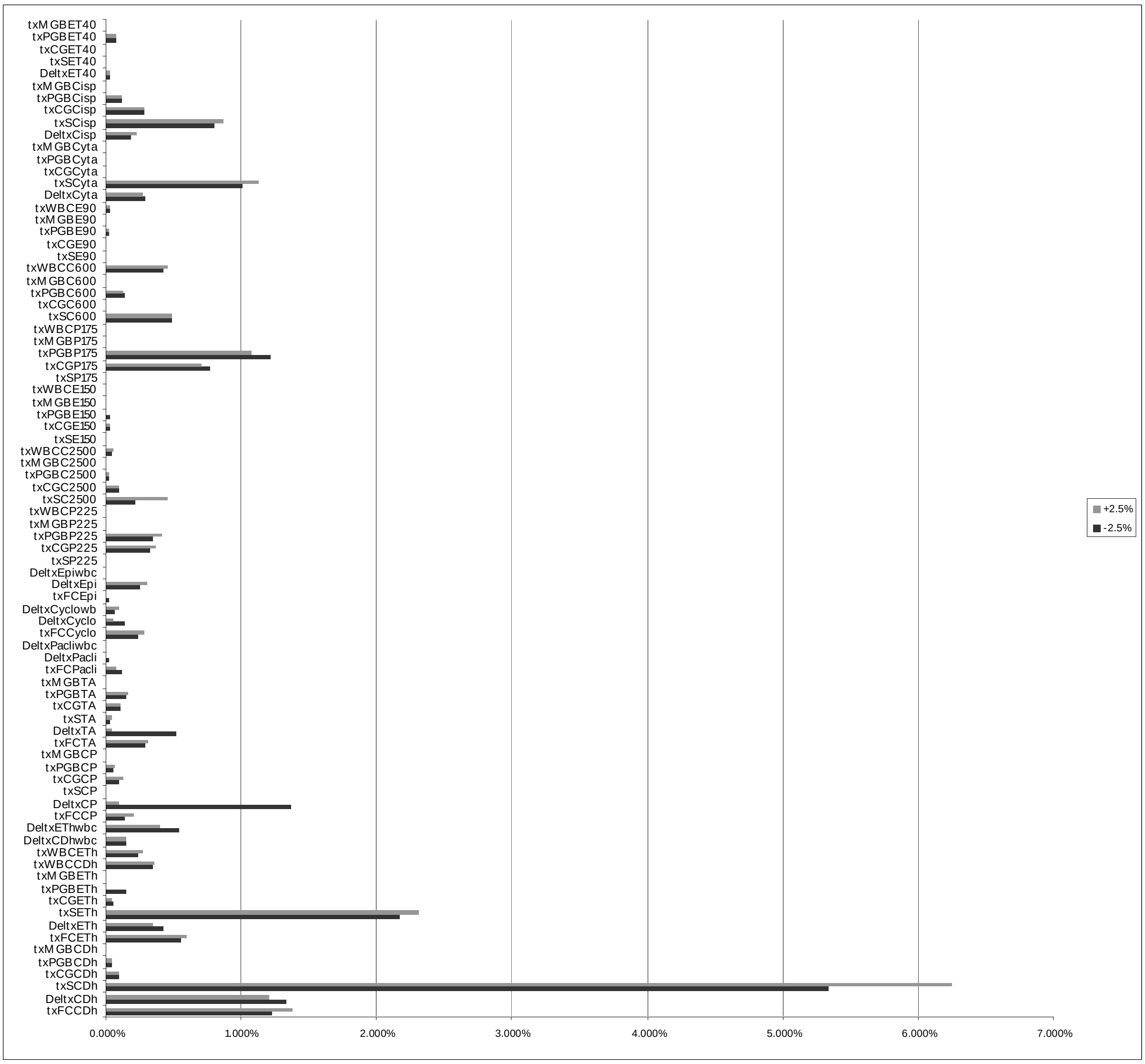

Figure A2: Sensitivity of chemotherapy parameters (part 2). The diagram depicts the change in the fitness value (%) after a parameter modification of 2.5%.

Further Simulation Results

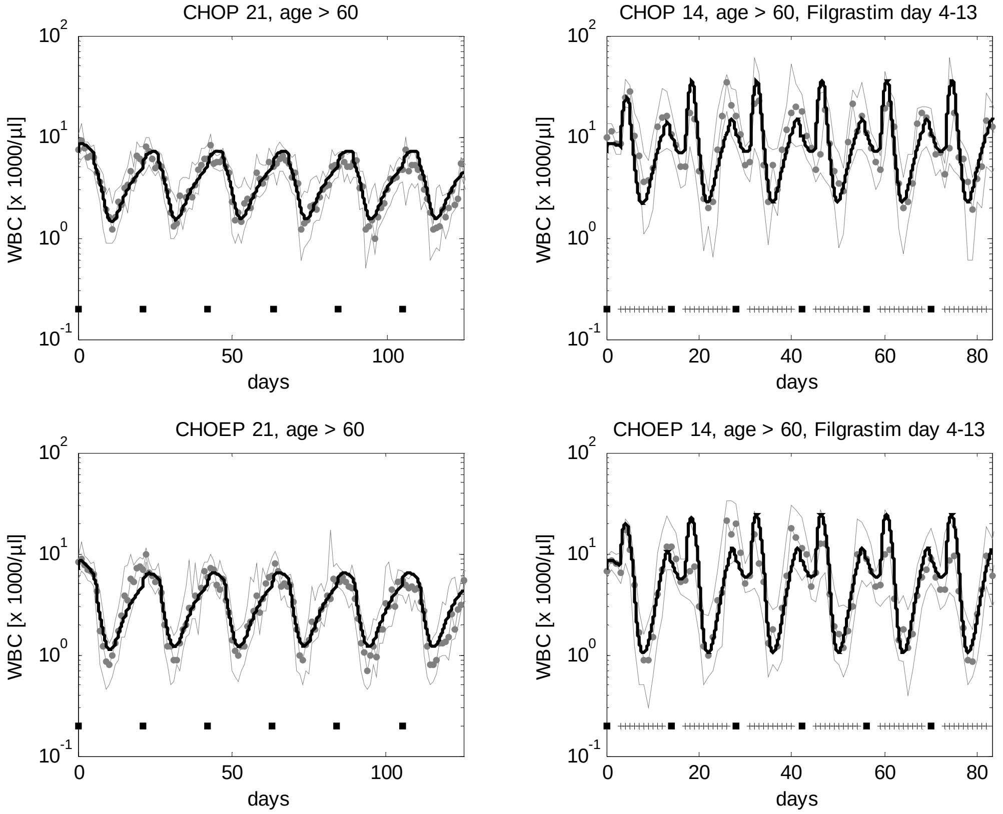

Figure A3: Simulation results for CHOP-21, CHOP-14, CHOEP-21 and CHOEP-14, elderly patients. The diagrams depict the simulated cell counts for CHOEP-14 and CHOEP-21 chemotherapy, (see also [1]), data: [2]. Dots represent patient medians, grey lines represent interquartile range of patient data, and squares represent the chemotherapy administrations.

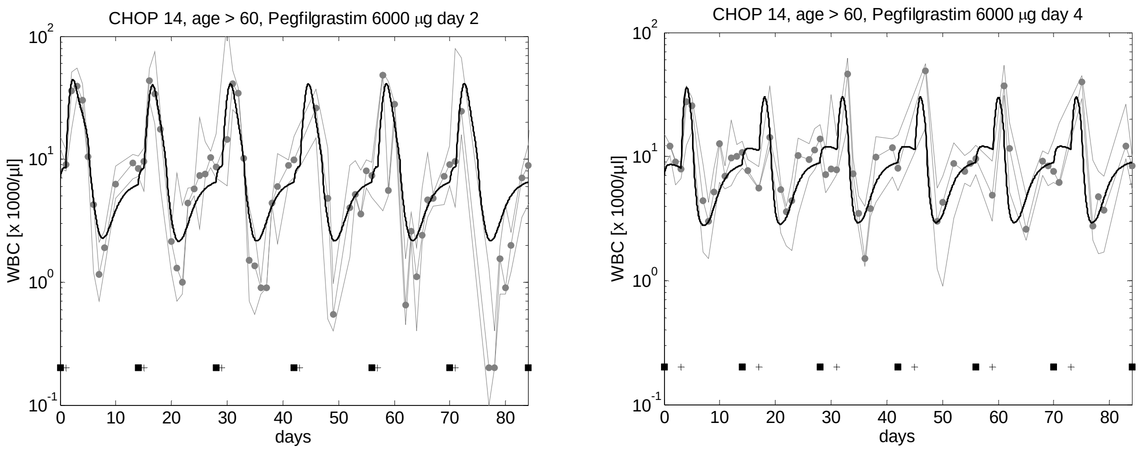

Figure A4: Simulation results for CHOP and Pegfilgrastim. The diagrams depict the simulated cell counts for CHOP chemotherapy with Pegfilgrastim 6000 µg on day 2 (left) and day 4 (data: [3]). Dots represent patient medians, grey lines represent interquartile range of

patient data, squares represent the chemotherapy administrations, + are G-CSF-injections (see also [1]).

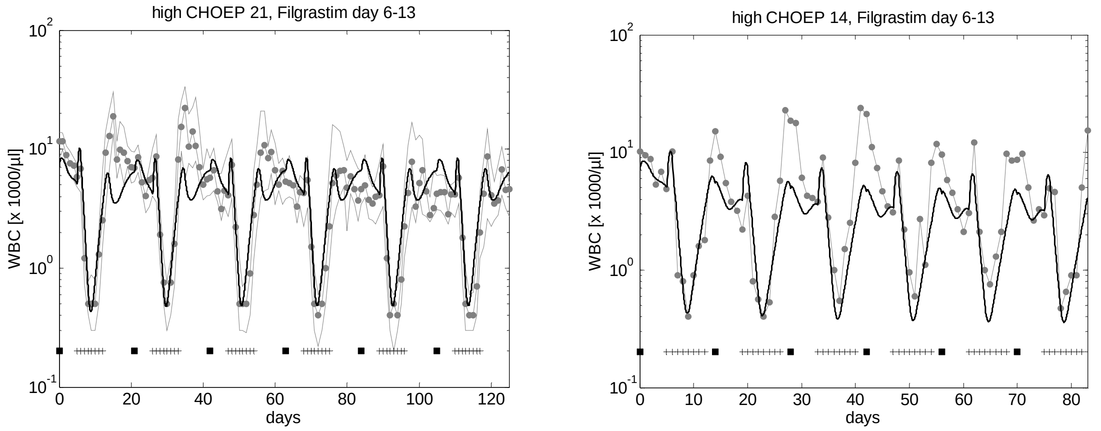

Figure A5: Simulation results of high CHOEP regimen. Simulated cell counts for high CHOEP-21 with Filgrastim 480 µg on days 6-13 (left); and high CHOEP-14 with Filgrastim on days 6-13 (right). Dots represent patient medians, grey lines represent interquartile range of patient data, squares represent the chemotherapy administrations, + are G-CSF-injections.

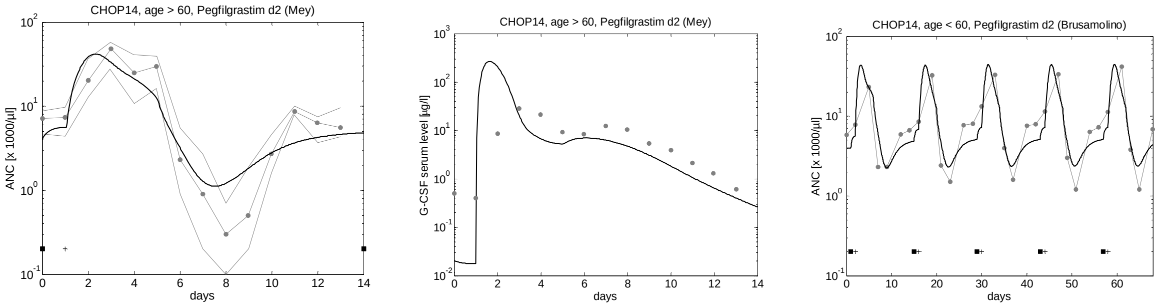

Figure A6: Simulation results for CHOP and Pegfilgrastim (literature data). The diagrams depict the simulated ANC counts or G-CSF serum concentrations for CHOP chemotherapy with Pegfilgrastim 6000 µg on day 2, data: [4, 5]. Dots represent patient data, grey lines represent interquartile range of patient data, squares represent the chemotherapy administrations, + are G-CSF-injections.

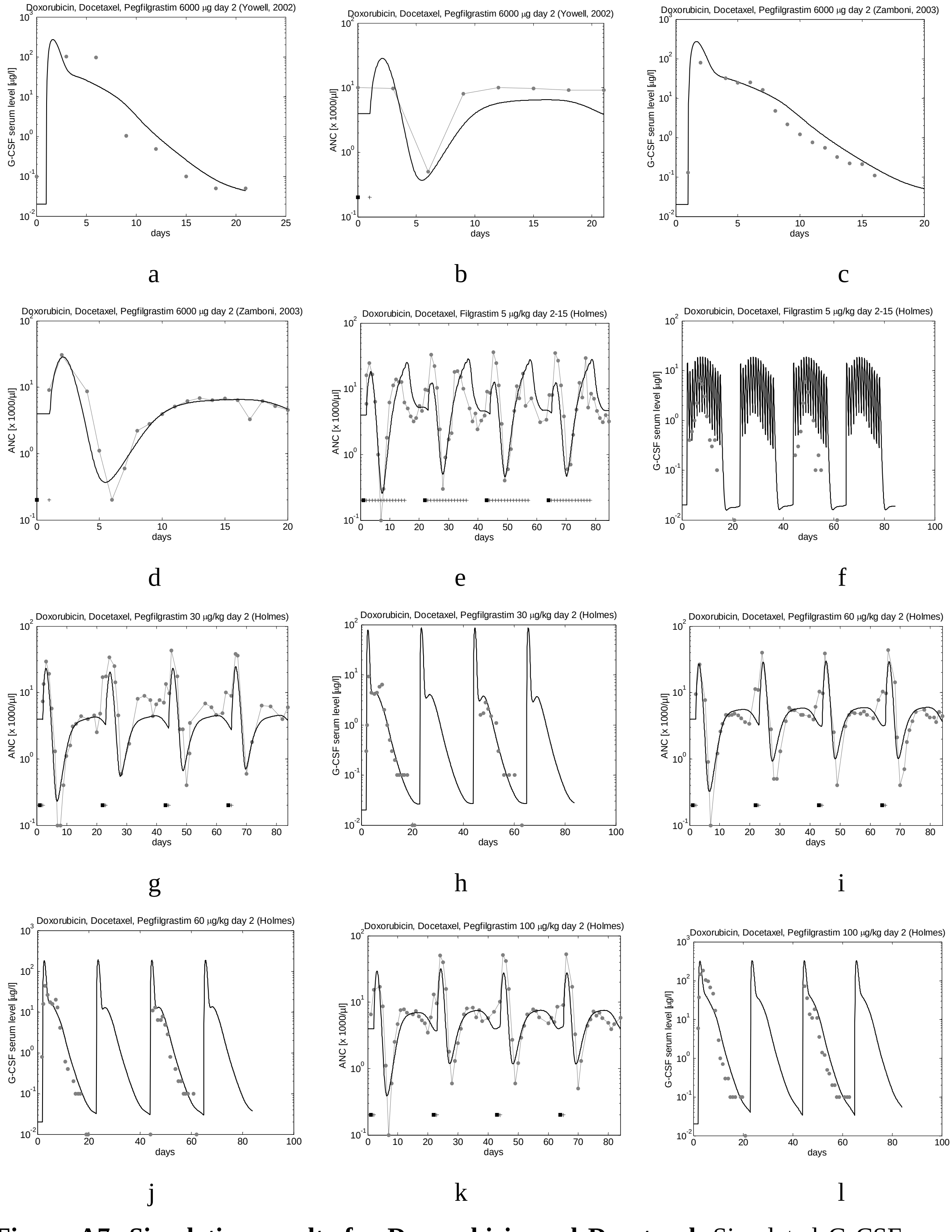

Figure A7: Simulation results for Doxorubicin and Docetaxel. Simulated G-CSF serum concentration and cell counts for Doxorubince and Docetaxel in breast cancer patients with Pegfilgrastim 6000 µg on day 2 (a, b), data: [6], (c, d), data: [7]; Filgrastim 5µg/kg on days 2-15 (e,f); and Pegfilgrastim 30µg/kg (g, h), 60µg/kg (i, j) or 100 µg/kg (k, l) on day 2. Dots represent patient medians, squares represent the chemotherapy administrations, + are G-CSF-injections, data: [8].

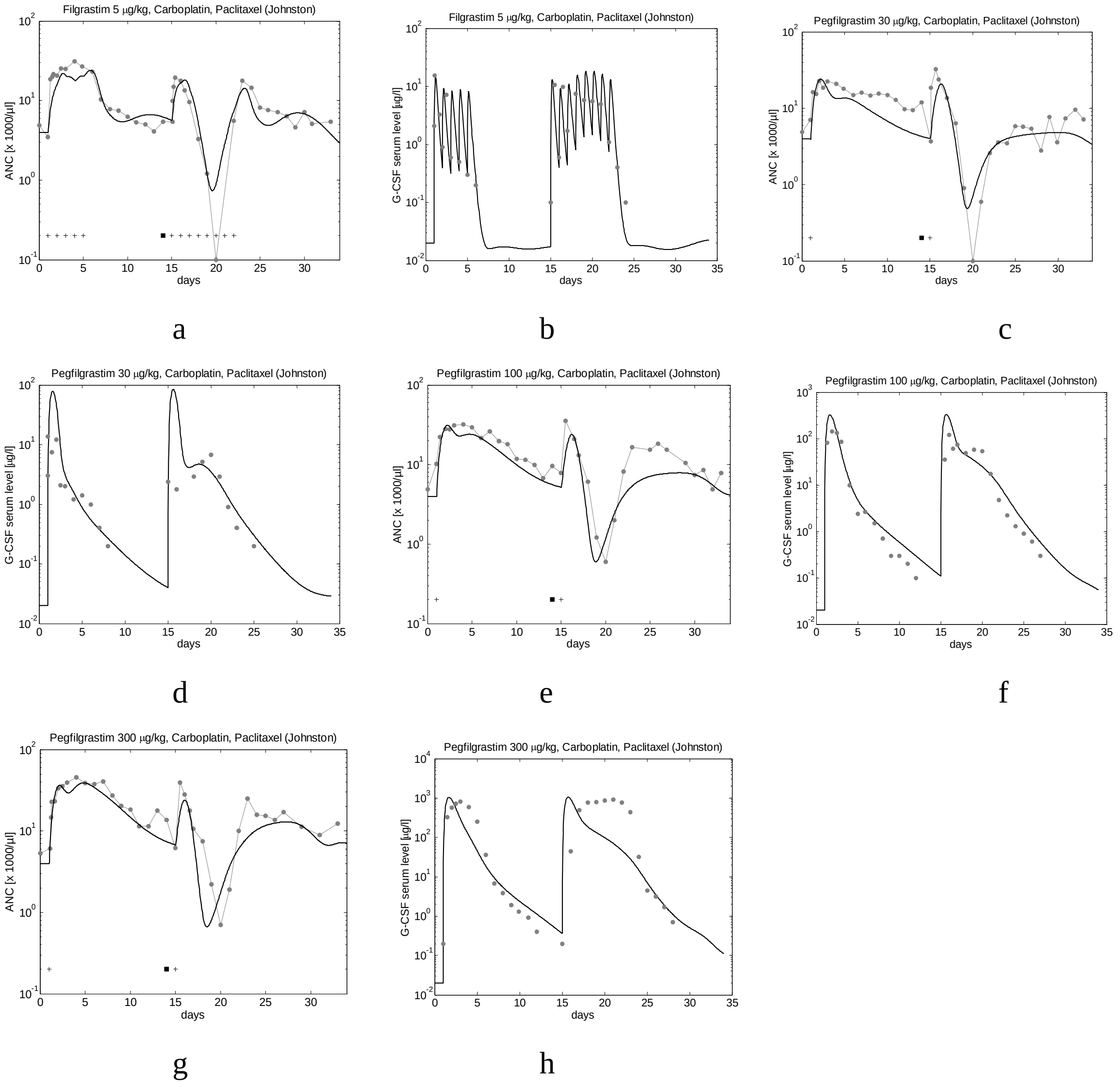

Figure A8: Simulation results for Carboplatin and Paclitaxel. Simulated G-CSF serum concentration and cell counts for with (a-b) Filgrastim 5µg/kg; (c-d) Pegfilgrastim 30 µg/kg on day 1; (e-f) Pegfilgrastim 100 µg/kg on day 1; and (g-h) Pegfilgrastim 300 µg/kg on day 1. Dots represent patient medians, squares represent the chemotherapy administrations, + are G-CSF-injections, data: [9].

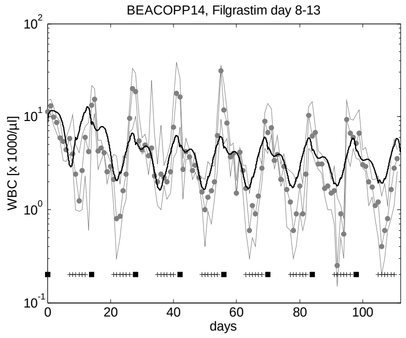

Figure A9: Simulation results for BEACOPP 14. Simulated cell counts for BEACOPP 14 with Filgrastim 480 µg on days 8-13. Dots represent patient medians, grey lines represent interquartile range of patient data, squares represent the chemotherapy administrations, + are G-CSF-injections.

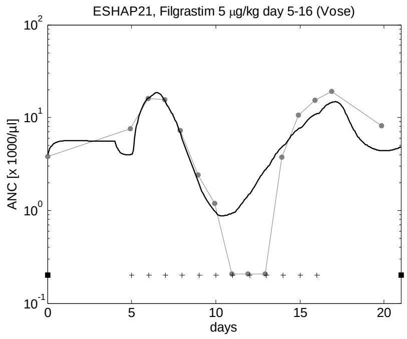

Figure A10: Simulation results for ESHAP. Simulated cell counts for ESHAP with Filgrastim 5µg/kg on days 5-16, data: [10]. Dots represent patient medians, grey lines represent interquartile range of patient data, squares represent the chemotherapy administrations, + are G-CSF-injections.

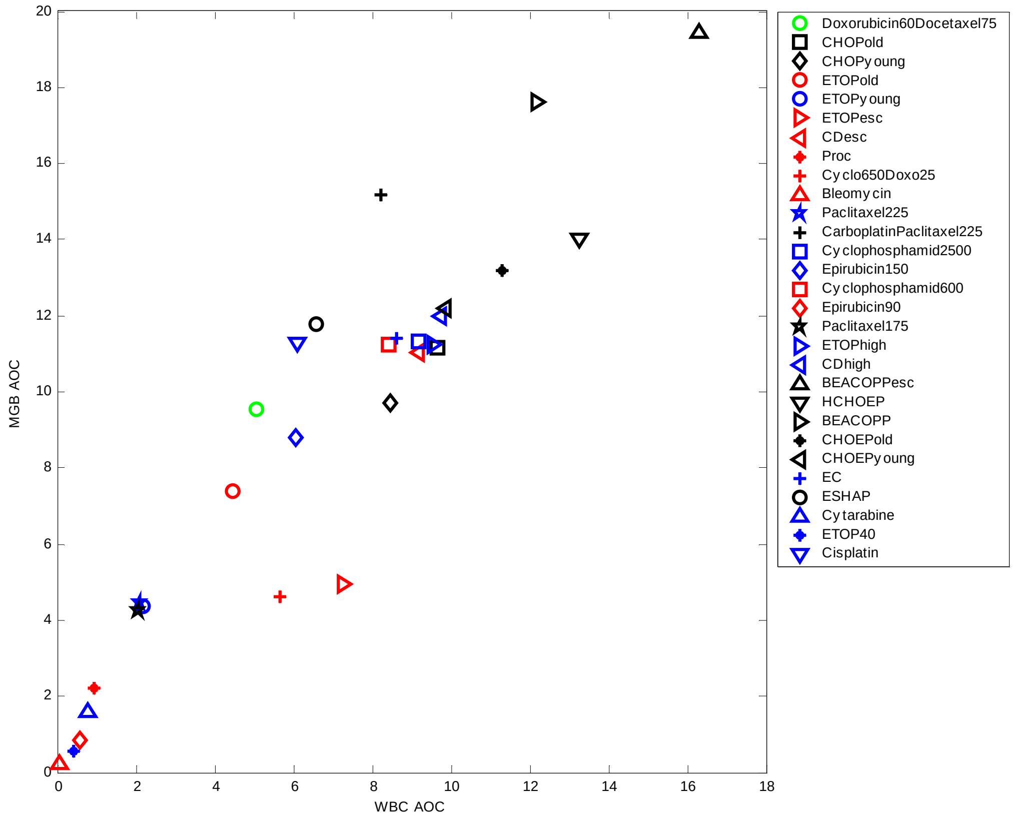

Figure A11: Correlation of MGB toxicity and peripheral toxicity. We determined cumulative toxicities of MGB and mature blood cells for each drug or drug combination considered. Toxicities are expressed in terms of AOC of normalized cell counts applying the steady-state value 1 as threshold. AOC is calculated over 28 days. Only a single injection of chemotherapy was simulated for this purpose. The unit of AOC is “d”. A good correlation between MGB toxicity and peripheral toxicity can be observed. Toxicity relations between schedules are plausible. Note that schedules may be attributed to different groups of patients.

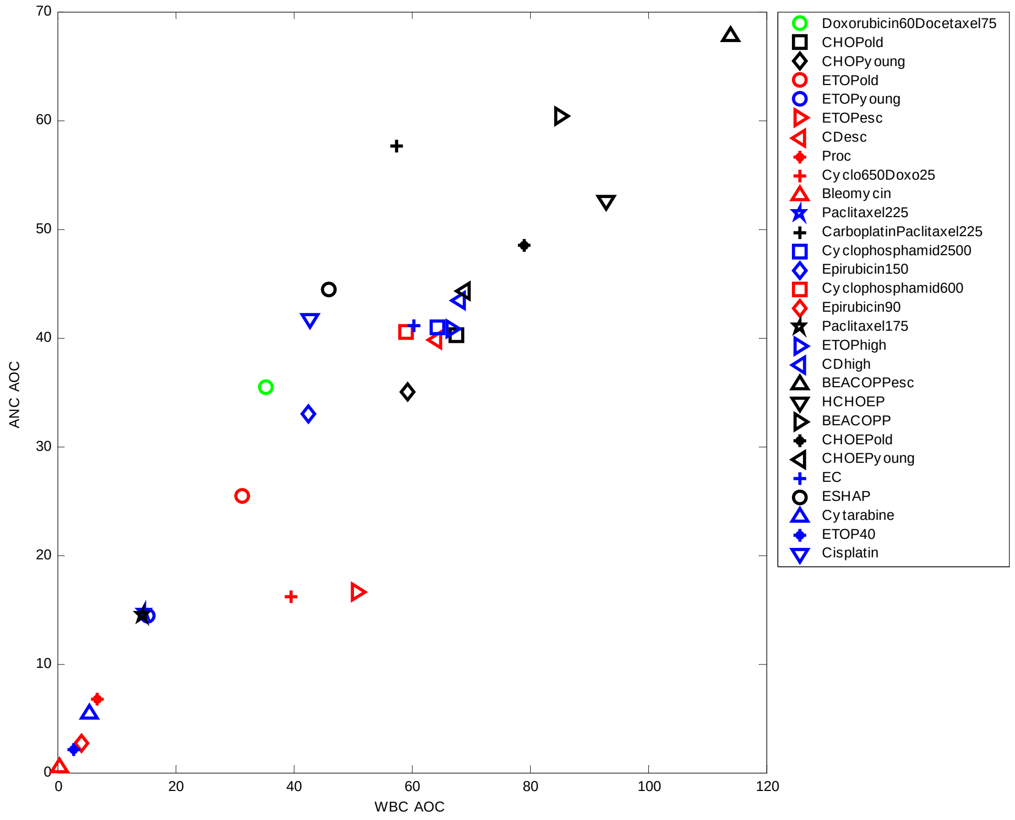

Figure A12: Correlation of granulocyte toxicity and leukocyte toxicity. We determined cumulative toxicities of MGB and mature blood cells for each drug or drug combination considered. Toxicities are expressed in terms of AOC of normalized cell counts applying the steady-state value 1 as threshold. AOC is calculated over 28 days. Only a single injection of chemotherapy was simulated for this purpose. The unit of AOC is “d”. A good correlation between granulocyte and leukocyte toxicity can be observed. Toxicity relations between schedules are plausible. Note that schedules may be attributed to different groups of patients.

References:

1. Scholz M, Schirm S, Wetzler M, Engel C, Loeffler M: **Pharmacokinetic and -dynamic modelling of G-CSF derivatives in humans**. *Theoretical biology & medical modelling* 2012, **9**:32.
2. Pfreundschuh M, Trumper L, Kloess M, Schmits R, Feller AC, Rube C, Rudolph C, Reiser M, Hossfeld DK, Eimermacher H *et al*: **Two-weekly or 3-weekly CHOP chemotherapy with or without etoposide for the treatment of elderly patients with aggressive lymphomas: results of the NHL-B2 trial of the DSHNHL**. *Blood* 2004, **104**(3):634-641.
3. Zwick C, Hartmann F, Zeynalova S, Poschel V, Nickenig C, Reiser M, Lengfelder E, Peter N, Schlimok G, Schubert J *et al*: **Randomized comparison of pegfilgrastim**

day 4 versus day 2 for the prevention of chemotherapy-induced leukocytopenia. *Annals of oncology : official journal of the European Society for Medical Oncology / ESMO* 2011, **22**(8):1872-1877.

4. Brusamolino E, Rusconi C, Montalbetti L, Gargantini L, Uziel L, Pinotti G, Fava S, Rigacci L, Pagnucco G, Pascutto C *et al*: **Dose-dense R-CHOP-14 supported by pegfilgrastim in patients with diffuse large B-cell lymphoma: a phase II study of feasibility and toxicity**. *Haematologica* 2006, **91**(4):496-502.
5. Mey UJ, Maier A, Schmidt-Wolf IG, Ziske C, Forstbauer H, Banat GA, Reber M, Strehl JW, Gorschlueter M: **Pegfilgrastim as hematopoietic support for dose-dense chemoimmunotherapy with R-CHOP-14 as first-line therapy in elderly patients with diffuse large B cell lymphoma**. *Supportive care in cancer : official journal of the Multinational Association of Supportive Care in Cancer* 2007, **15**(7):877-884.
6. Yowell SL, Blackwell S: **Novel effects with polyethylene glycol modified pharmaceuticals**. *Cancer treatment reviews* 2002, **28**:3-6.
7. Zamboni WC: **Pharmacokinetics of pegfilgrastim**. *Pharmacotherapy* 2003, **23**(8 Pt 2):9S-14S.
8. Holmes FA, Jones SE, O'Shaughnessy J, Vukelja S, George T, Savin M, Richards D, Glaspy J, Meza L, Cohen G *et al*: **Comparable efficacy and safety profiles of once-per-cycle pegfilgrastim and daily injection filgrastim in chemotherapy-induced neutropenia: a multicenter dose-finding study in women with breast cancer**. *Annals of oncology : official journal of the European Society for Medical Oncology / ESMO* 2002, **13**(6):903-909.
9. Johnston E, Crawford J, Blackwell S, Bjurstrom T, Lockbaum P, Roskos L, Yang BB, Gardner S, Miller-Messana MA, Shoemaker D *et al*: **Randomized, dose-escalation study of SD/01 compared with daily filgrastim in patients receiving chemotherapy**. *J Clin Oncol* 2000, **18**(13):2522-2528.
10. Vose JM, Crump M, Lazarus H, Emmanouilides C, Schenkein D, Moore J, Frankel S, Flinn I, Lovelace W, Hackett J *et al*: **Randomized, multicenter, open-label study of pegfilgrastim compared with daily filgrastim after chemotherapy for lymphoma**. *J Clin Oncol* 2003, **21**(3):514-519.
